# Human Adenovirus 52 Uses Sialic Acid-containing Glycoproteins and the Coxsackie and Adenovirus Receptor for Binding to Target Cells

**DOI:** 10.1371/journal.ppat.1004657

**Published:** 2015-02-12

**Authors:** Annasara Lenman, A. Manuel Liaci, Yan Liu, Carin Årdahl, Anandi Rajan, Emma Nilsson, Will Bradford, Lisa Kaeshammer, Morris S. Jones, Lars Frängsmyr, Ten Feizi, Thilo Stehle, Niklas Arnberg

**Affiliations:** 1 Division of Virology, Department of Clinical Microbiology, Umeå University, Umeå, Sweden; 2 University of Tübingen, Interfaculty Institute of Biochemistry, Tübingen, Germany; 3 Glycosciences Laboratory, Department of Medicine, Imperial College London, London, United Kingdom; 4 Division of Infectious Diseases, Naval Medical Center, San Diego, California, United States of America; 5 Vanderbilt University School of Medicine, Nashville, Tennessee, United States of America; Stony Brook University, UNITED STATES

## Abstract

Most adenoviruses attach to host cells by means of the protruding fiber protein that binds to host cells via the coxsackievirus and adenovirus receptor (CAR) protein. Human adenovirus type 52 (HAdV-52) is one of only three gastroenteritis-causing HAdVs that are equipped with two different fiber proteins, one long and one short. Here we show, by means of virion-cell binding and infection experiments, that HAdV-52 can also attach to host cells via CAR, but most of the binding depends on sialylated glycoproteins. Glycan microarray, flow cytometry, surface plasmon resonance and ELISA analyses reveal that the terminal knob domain of the long fiber (52LFK) binds to CAR, and the knob domain of the short fiber (52SFK) binds to sialylated glycoproteins. X-ray crystallographic analysis of 52SFK in complex with 2-*O*-methylated sialic acid combined with functional studies of knob mutants revealed a new sialic acid binding site compared to other, known adenovirus:glycan interactions. Our findings shed light on adenovirus biology and may help to improve targeting of adenovirus-based vectors for gene therapy.

## Author Summary

HAdVs are common pathogens in humans, causing disease mainly in eyes, airways and gastrointestinal tract. Most HAdVs are equipped with twelve protruding fiber proteins that mediate attachment to host cell receptor molecules. Recently, a new human gastroenteritis-associated adenovirus (HAdV-52) was identified and classified as the first member of a novel species (HAdV-G). Unlike most other HAdVs, this virus contains two different fiber proteins, a long and a short one, a feature shared only with the two members of species HAdV-F (HAdV-40 and -41). To gain further insights into the mechanisms of HAdV-52 infection of human cells, we set out to identify the host cell receptors used by the long and short fibers. We find that the long fiber binds to a protein-based receptor known as the coxsackievirus and adenovirus receptor (CAR), and that the short fiber binds to glycoproteins that contain sialic acid-capped glycans. The crystal structure determination of a complex of the short fiber knob bound to sialic acid demonstrates that this interaction is unique among HAdVs, and bioinformatic analysis indicates that simian AdVs may also engage sialic acids in the manner seen in HAdV-52. The results presented here provide insights into the plasticity of adenovirus-host cell interactions.

## Introduction

Human adenoviruses (HAdVs) are classified into seven species (A-G), with more than 50 different types known to date [1]. Most HAdVs cause disease in the eyes (members of species HAdV-B, -D, -E), airways (HAdV-A, -B, -C, -E) and gastrointestinal tract (HAdV-F mainly) [2]. HAdV-52 was recently identified as a novel, human pathogen associated with gastroenteritis [3], and was found to be divergent from other HAdVs placing this virus in a new species (HAdV-G). HAdVs from species HAdV-A and HAdV-C through HAdV-F use the coxsackievirus and adenovirus receptor (CAR) as a primary adhesion receptor [4–6]. Members of species HAdV-B that cause ocular, respiratory and/or urinary tract infections utilize CD46 and/or desmoglein-2 as cellular receptors [7–10]. Specific members of species HAdV-D cause a more severe ocular infection, epidemic keratoconjunctivitis, and engage glycoproteins that carry glycans mimicking those in the GD1a ganglioside: Neu5Acα(2–3)Galβ(1–3)GalNAcβ(1–4)(Neu5Acα(2–3))Galβ(1–4)Glc as receptors [11–13]. In addition to these AdVs, canine AdV-2 (species CAdV-A) is another glycan-binding AdV, engaging Neu5Acα(2–3)[6S]Galβ(1–4)GlcNAc-containing glycans at a different location on the knob[14]. The locations of the two known glycan binding sites are distinct from the regions that allow some knobs to engage CAR [15] or CD46 [16]. In the case of CAR, the length and flexibility of the fiber shaft also seem to play a role in infection, as short and sturdy fibers cannot bend to bind CAR on a cell surface [17]. HAdVs can also enter cells through interactions with coagulation factors that mediate indirect binding to heparan sulfate proteoglycans on target cells [18,19]. With a few exceptions, HAdVs are equipped with a single type of capsid fiber protein that interacts with receptors via its knob domain. HAdV-40, -41, and -52 on the other hand are equipped with two different fibers, one long and one short [20,21]. The long fibers of HAdV-40 and -41 bind to CAR [6], but no function has been described for any of the short fibers. Phylogenetic analyses have shown that the closest human relatives to the knobs of the HAdV-52 long and short fibers are the knobs of the long and short fibers of species HAdV-F (HAdV-40 and -41) [22].

Adenoviruses are frequently used as vectors for diverse applications including vaccination [23–25], treatment of cancer [26] and hereditary disorders [27], cardiovascular applications [28], and stem cell research [29]. Three of the main challenges for the most commonly used HAdV-5 (species HAdV-C) based vectors are: i) pre-existing, neutralizing antibodies [30], ii) poor access to CAR [31], and, iii) coagulation factor-dependent off-target transduction of the liver [18]. Potential solutions to these obstacles have been to use vector candidates based on less common HAdV types [24], and HAdV types that use receptors alternative to CAR [32,33]. Inefficient targeting have been addressed by ablating CAR- and/or coagulation factor-interactions, and/or by retargeting to receptors that are overexpressed on target cells [34]. Ideally, a multi-purpose vector would therefore be based on a rare type that efficiently target host cells by means of specific receptor interactions and low or absent off-target transduction.

The seroprevalence for HAdV-40 and -41 is relatively high (40–50%) in the human population [35–37]. The seroprevalence for HAdV-52 in humans has not been investigated, but the close relationship with simian AdVs and the low frequency of detection in humans [3,38] suggest that the seroprevalence in humans is low. In combination with its uncommon capsid organization this prompted us to gain more insight into HAdV-52 interactions with host cells and more specifically to identify cellular receptors used by HAdV-52 for attachment to host cells.

## Results and Discussion

### HAdV-52 binds both to CAR and sialic acid-containing glycans on target cells

**Fig 1:**
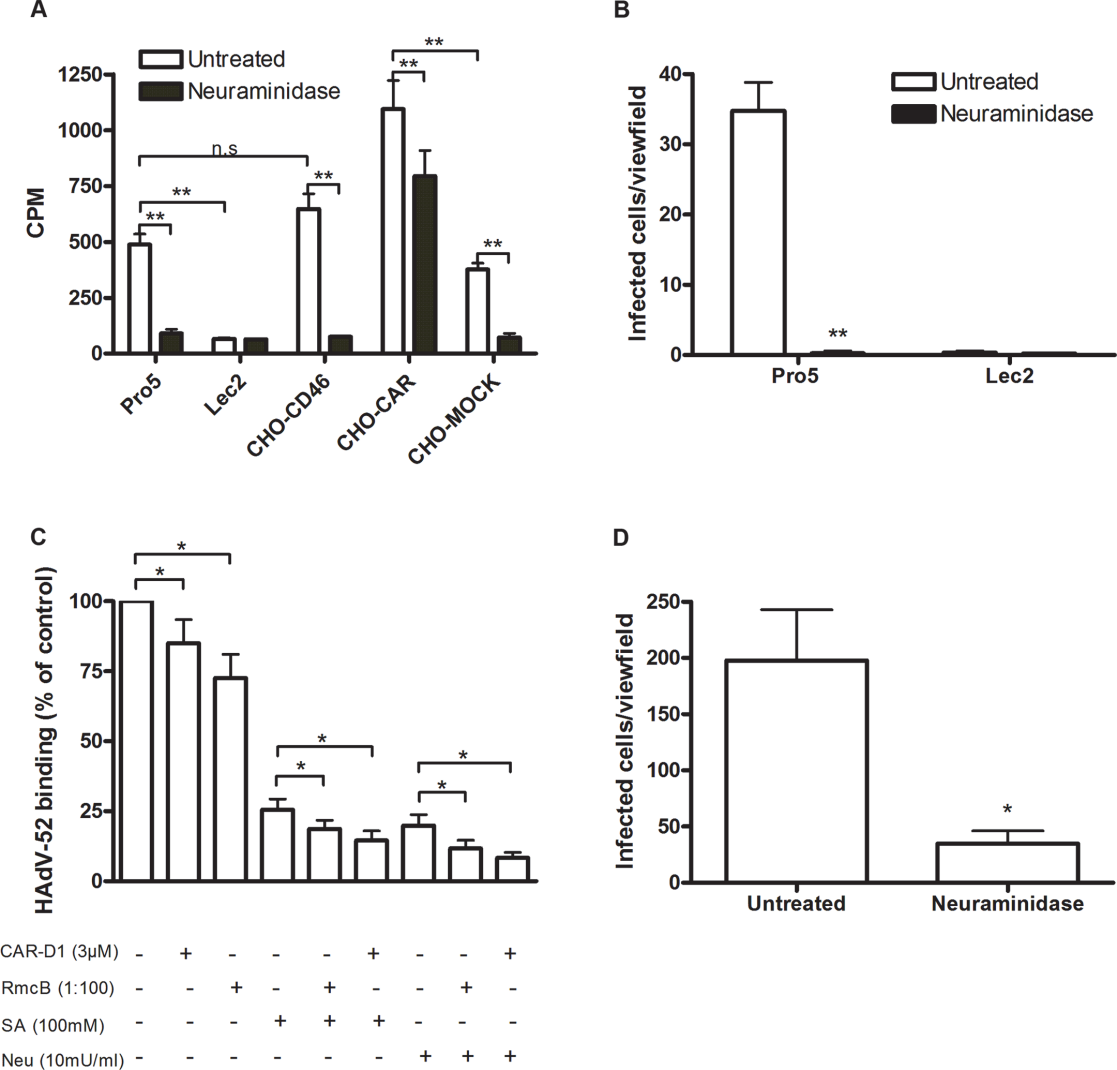
HAdV-52 uses sialic acid and CAR for binding to and infection of cells. (A) ^35^S-labeled HAdV-52 virion binding to CHO cells expressing or lacking known HAdV receptors. Pro-5 is a sialic acid-positive, reference cell line and parental cell line to sialic acid-negative Lec2 cells. CHO-CD46 and CHO-CAR cells express human CD46 and CAR, respectively. CHO-MOCK is mock transfected with respect to CHO-CAR. Black bars show HAdV-52 binding to cells after pretreatment with *V*. *cholerae* neuraminidase. Binding was quantified by liquid scintillation counting and shown as counts per minute (CPM). (B) HAdV-52 infection of Pro-5 and Lec2 pretreated with (black bars) or without (white bars) *V*. *cholerae* neuraminidase. The number of infected cells was quantified by immunofluorescence. (C) ^35^S-labeled HAdV-52 virion binding to A549 cells. Virions were preincubated with or without soluble CAR-D1 or sialic acid monosaccharides, and cells were preincubated with or without mouse anti-CAR mab (clone RmcB) or *V*. *cholerae* neuraminidase as indicated, prior to binding. (D) HAdV-52 infection of A549 cells pretreated with or without *V*. *cholerae* neuraminidase. All experiments were performed three times with duplicate samples in each experiment. Error bars represent mean ± SD. n.s = not significant, * P of < 0.05 and ** P of < 0.01.

**Fig 2:**
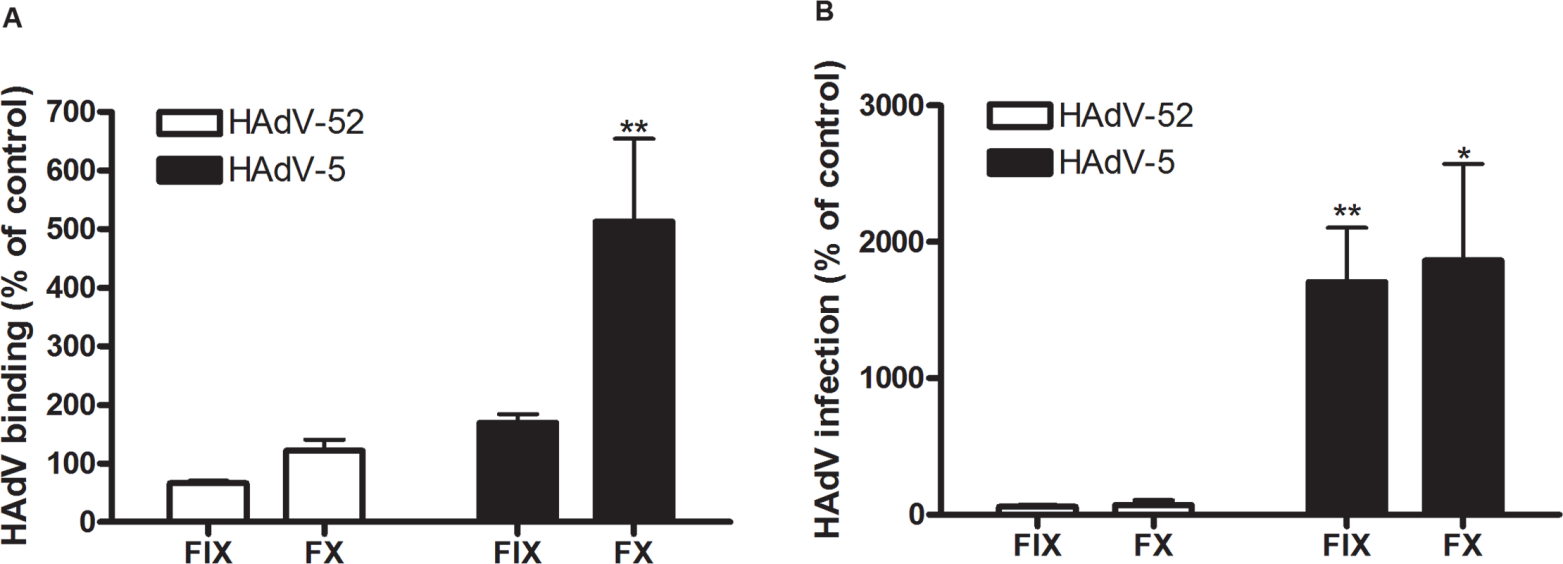
HAdV-52 does not use coagulation factors for binding and infection of A549 cells. (A) ^35^S-labeled HAdV-52 virion binding to A549 cells after virion preincubation with physiological concentrations of coagulation factor IX and X (FIX: 5μg/ml and FX: 10μg/ml). (B) HAdV-52 infection of A549 cells after virion preincubation with physiological concentrations of coagulation factors. All experiments were performed three times with duplicate samples in each experiment. Error bars represent mean ± SD. * P of < 0.05 and ** P of < 0.01 versus control.

To investigate whether CAR, CD46 or sialic acid-containing glycans can function as receptors for HAdV-52, we first analyzed ^35^S-labelled HAdV-52 virion binding to CHO cells expressing or lacking these receptors. HAdV-52 bound with similar efficiency to sialic acid-expressing control CHO (Pro-5) cells, CD46-expressing CHO cells and CHO MOCK (with respect to CAR), but with increased efficiency to CAR-expressing CHO cells and with decreased efficiency to sialic acid-lacking Lec2 cells (derived from Pro-5) as compared to the other cells (**Fig. 1A**). Pretreatment of cells with sialic acid-cleaving *V*. *cholerae* neuraminidase reduced HAdV-52 binding to background levels for all cells except to CHO-CAR. To test if this neuraminidase removed sialic acids with equal efficiency from all cells, we treated the cells with *V*. *cholerae* neuraminidase and quantified MAL-II lectin binding. This treatment reduced MAL-II binding to background levels **(S1 Fig.**) and we therefore concluded that HAdV-52 could bind to CHO-CAR independently of sialic acid. As HAdV-52 bound with equal efficiency to Pro-5, CHO-MOCK, and CHO-CD46, and as neuraminidase treatment of CHO-CD46 cells reduced HAdV-52 binding efficiently, these results indicate that CD46 is probably of no or low importance as a receptor for HAdV-52. HAdV-52 also infected Pro-5 cells more efficiently than Lec2 cells, and pretreatment of Pro-5 cells with neuraminidase abolished infection (**Fig. 1B**). HAdV-52 is associated with gastroenteritis, but the number of human cases described is limited and the cellular tropism of the virus is unclear. We therefore investigated the relative contributions of sialic acid and CAR using respiratory A549 cells, which support productive infection of most HAdVs and express both sialic acid and CAR at the cell surface. HAdV-52 binding to these cells was reduced by 20% and 25%, respectively, when preincubating HAdV-52 virions with soluble CAR-D1 (consisting of the N-terminal, most membrane-distal immunoglobulin-like domain), or when preincubating cells with monoclonal anti-CAR antibodies (clone RmcB) prior to virion binding (**Fig. 1C**). CAR-D1 and anti-CAR antibodies reduced HAdV-5 binding with 50% and 75%, respectively **(S2 Fig.**), thus demonstrating their function. On the other hand, HAdV-52 binding was reduced by 75% and 80% after preincubating virions with sialic acid or when pretreating cells with neuraminidase, respectively, prior to virion binding. Pretreatments with CAR-D1 or anti-CAR antibodies in combination with either sialic acid or neuraminidase reduced binding to background levels. The involvement of sialic acid-containing glycans as functional human cell receptors for HAdV-52 was confirmed by neuraminidase pretreatment of A549 cells, which reduced HAdV-52 infection by at least 80% (**Fig. 1D**). Finally, preincubation of virions with coagulation factor IX and X efficiently enhanced HAdV-5 binding to and infection of A549 cells but had no or limited effect on HAdV-52 (**Fig. 2A,B**). These results show that HAdV-52 does not use FIX, FX, or CD46 for attachment to A549 cells. We conclude that HAdV-52 binds to A549 cells mainly via sialic acid-containing glycans, and that the role of CAR is dwarfed by that of the sialylated receptors. However, we cannot exclude that the role of CAR as an attachment receptor for HAdV-52 may be more pronounced on other cell types than on A549 cells.

### The short fiber of HAdV-52 binds to sialic acid and the long fiber binds to CAR

**Fig 3:**
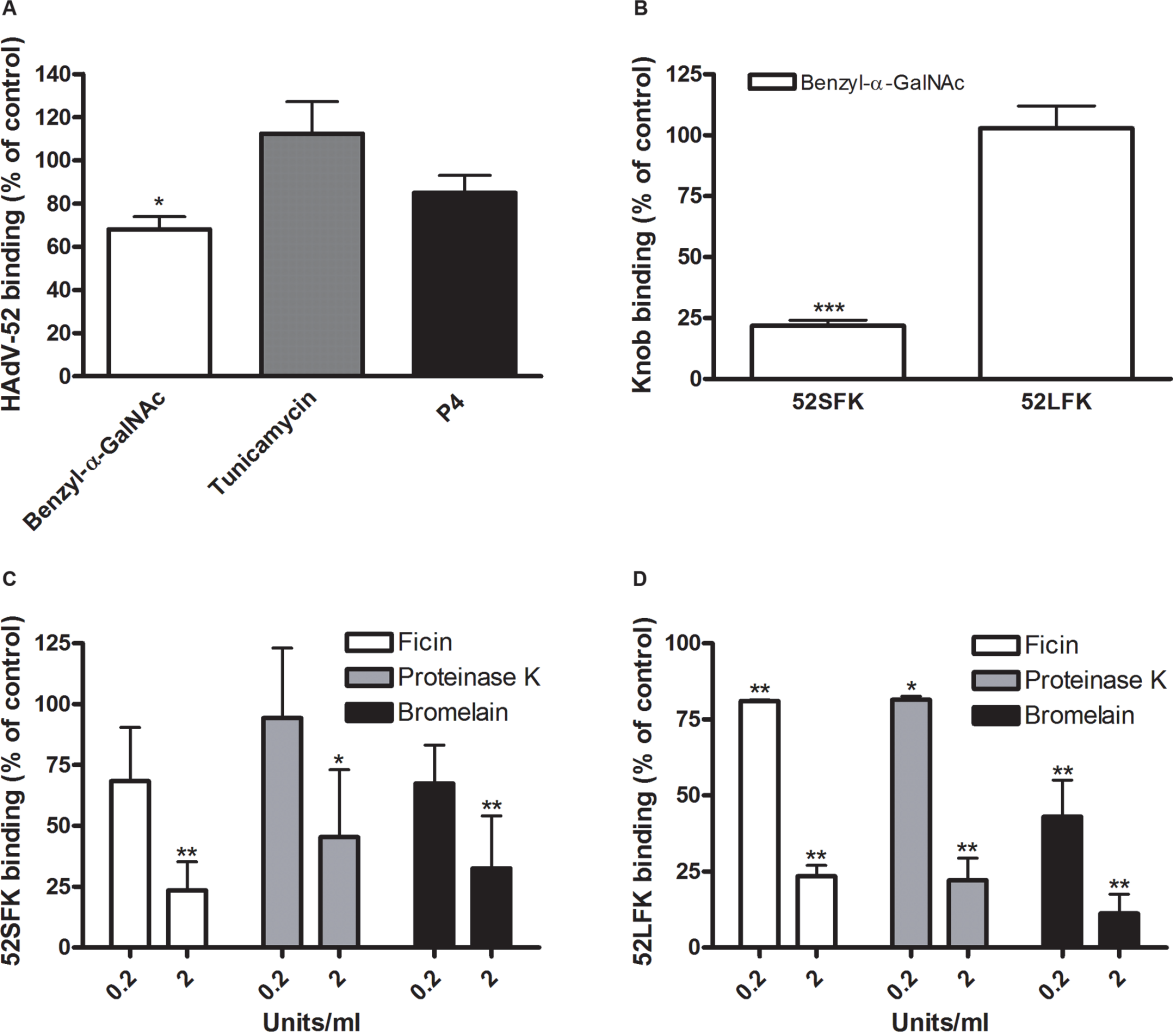
HAdV-52 short fiber knob binds to *O*-linked glycoproteins on A549 cells. (A) ^35^S-labeled HAdV-52 virion binding to A549 cells pretreated with benzyl-α-GalNAc, tunicamycin or P4 (inhibitors of *O*-linked glycan synthesis, *N*-linked glycan synthesis, and glycolipid synthesis, respectively). (B) HAdV-52 short fiber knobs (52SFK) and HAdV-52 long fiber knobs (52LFK) binding to A549 cells pretreated with benzyl-α-GalNAc (inhibitor of *O*-linked glycan synthesis). (C) 52SFK and (D) 52LFK binding to A549 cells pretreated with ficin, proteinase K or bromelain proteases at indicated concentrations. All experiments were performed three times with duplicate samples in each experiment. Error bars represent mean ± SD. * P of < 0.05, ** P of < 0.01 and *** P of < 0.001 versus control.

To characterize the nature of the sialic acid-containing glycans as receptors and the mechanism of interaction, we next quantified binding of HAdV-52 virions and HAdV-52 long and short fiber knobs (52LFK and 52SFK) to A549 cells pretreated with enzymes, lectins or metabolic inhibitors that alter the expression levels of cell surface molecules. Whereas inhibitors of glycolipid biosynthesis (P4) and *N-* (via Asp) linked glycosylation (tunicamycin) did not reduce virion binding to A549 cells significantly (**Figs. 3A, S3A,B**), benzyl *N*-acetyl-α-D-galactosaminide (benzyl-α-GalNAc, an inhibitor of *O*-linked glycosylation, via Ser or Thr) reduced binding of both the virions and 52SFK, but not of 52LFK (**Fig. 3A,B**). Protease (ficin, proteinase K, and bromelain) treatments of the same cells reduced binding of both 52SFK and 52LFK by 55–85% (**Fig. 3C,D**). These results suggest that on A549 cells, in contrast with the 52LFK which engages proteins directly without involvement of glycans, mucin type *O*-linked glycans are the dominant receptors for 52SFK and glycolipids and *N*-linked glycans appear not to play a major role.

**Fig 4:**
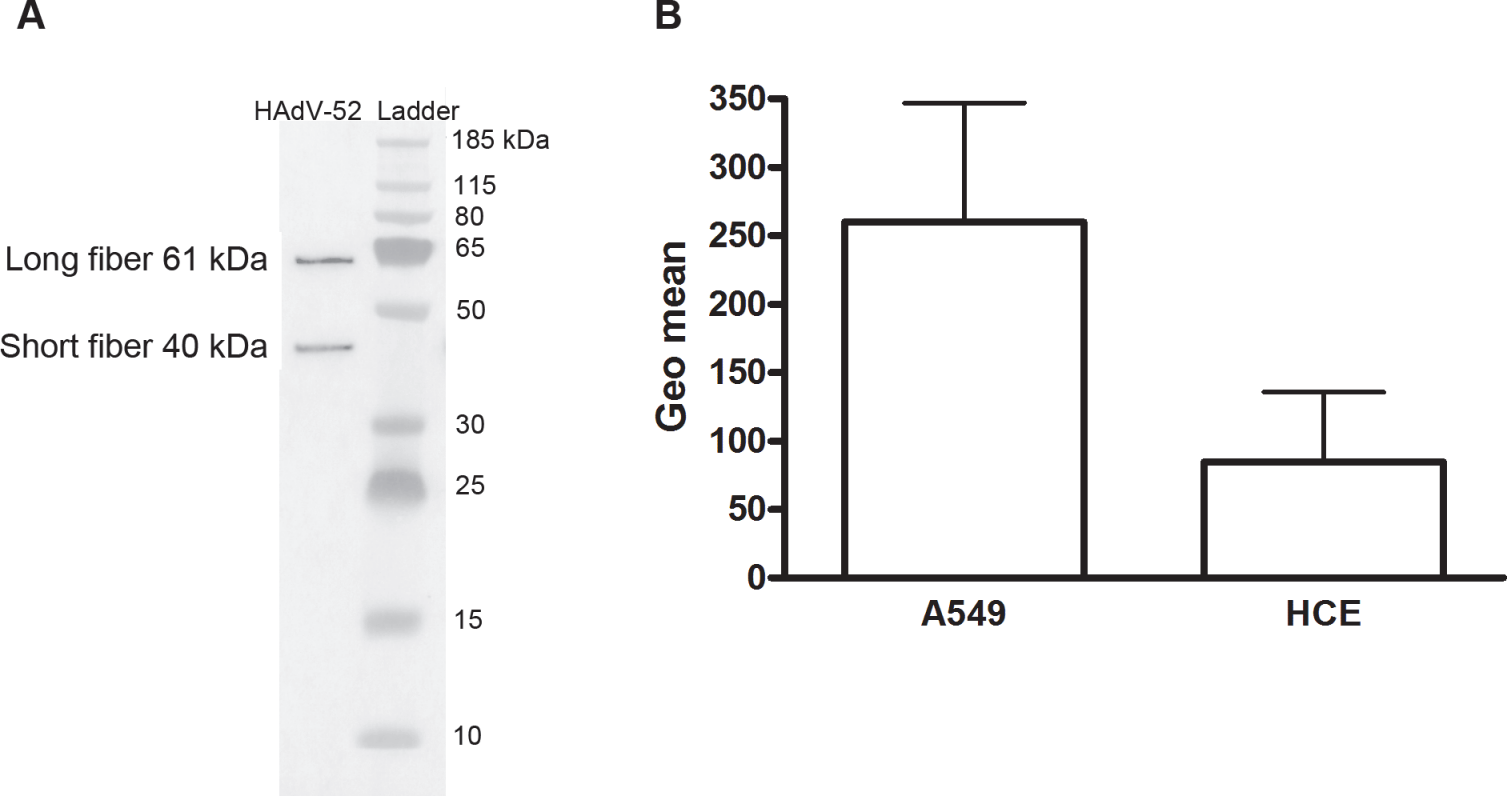
Virion composition and relative expression of CAR and sialic acid on human epithelial cells. A) Western blot analysis of HAdV-52 virion fiber content using a mouse mab (clone 4D2) recognizing an epitope (MKRARPSEDTFNPVYPY) conserved in the tail domain of all HAdVs. The experiment was performed three times (with three different virus preparations) and the figure shows one representative set of results. B) Flow cytometry analysis of CAR expression on A549 and human corneal epithelial (HCE) cells using an anti CAR mouse mab (clone E1-1). Data are shown as geometrical mean (geo mean) and the experiment was performed three times with duplicate samples in each experiment. Error bars represent means ± SD.

**Fig 5:**
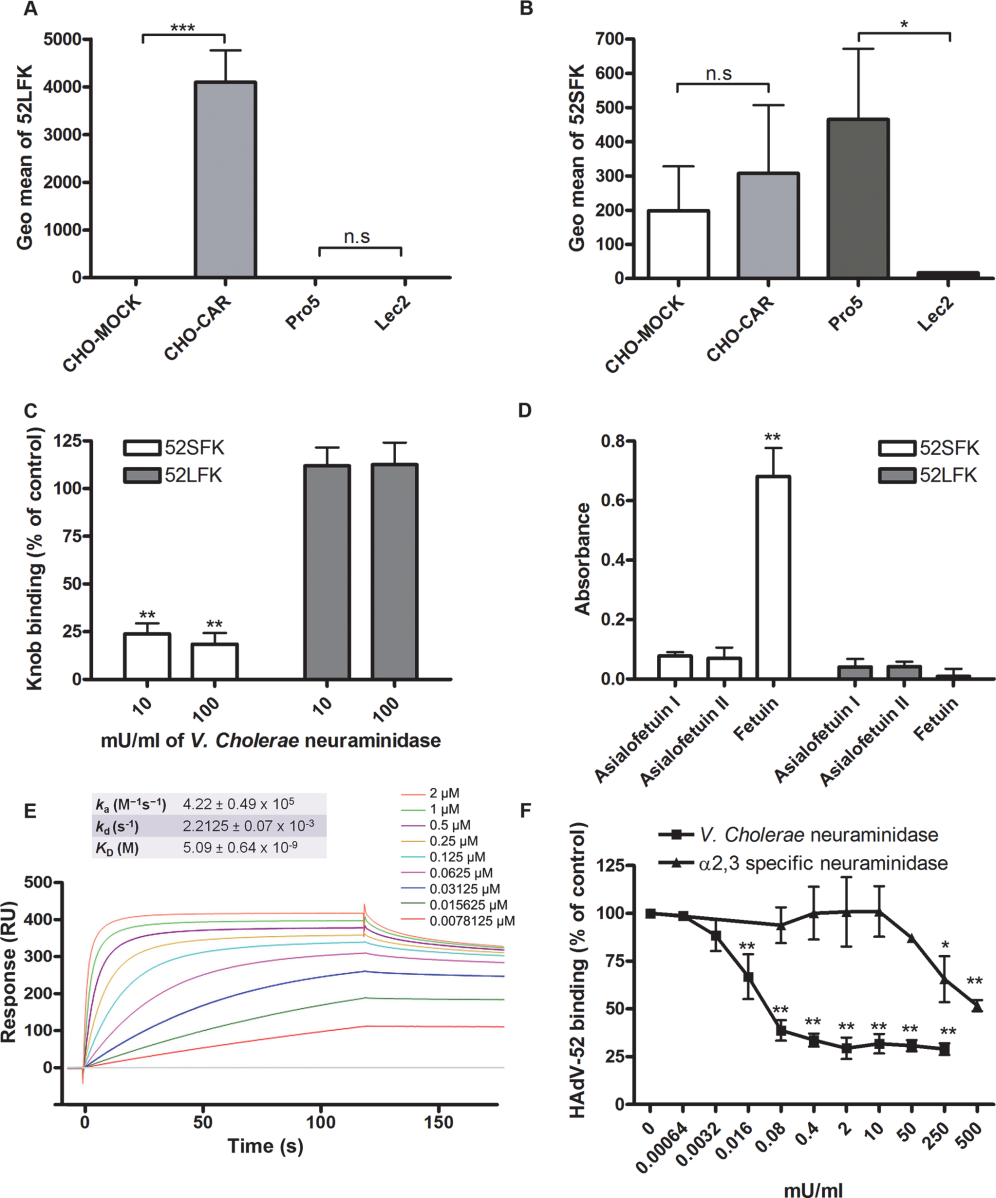
HAdV-52 short fiber knob binds to sialic acid and long fiber knob binds to CAR. (A) HAdV-52 long fiber knob (52LFK) and (B) HAdV-52 short fiber knob (52SFK) binding to CHO-cells lacking human CAR (all cells except CHO-CAR), lacking sialic acid (only Lec2) and expressing human CAR (CHO-CAR). (C) 52SFK and 52LFK binding to A549 cells pretreated with *V*. *cholerae* neuraminidase. (D) ELISA analysis of 52SFK and 52LFK (in solution) binding to immobilized, sialylated fetuin and asialofetuin type I (chemically prepared) and II (enzymatically prepared). Relative absorbance is shown. (E) Surface plasmon resonance analysis of 52LFK (in solution) binding to CAR (immobilized). A twofold dilution series of 52LFK is shown, ranging from 2 μM to 8 nM. Results are shown as response units (RU). (F) ^35^S-labeled HAdV-52 virion binding to A549 cells pretreated with *V*. *cholerae* neuraminidase (removes α2,3-, α2,6- and α2,8-linked sialic acid) or α2,3-specific neuraminidase at indicated concentrations. All experiments were performed three times with duplicate samples in each experiment. Figure E show one representative set of results. Error bars represent mean ± SD, n.s = not significant, * P of < 0.05, ** P of < 0.01 and *** P of < 0.001 versus control.

To determine the relative contribution of each fiber to cell attachment and infectivity, we first performed western blot analysis to characterize the relative fiber content in virus particles. Unlike HAdV-41 virions, which contain short and long fibers in a 6:1 ratio [39], HAdV-52 virions contained equal amounts of long and short fibers according to western blot analysis using a monoclonal antibody, which recognizes an epitope that is conserved in all HAdVs (**Fig. 4A**). This suggests that the apparent key role of sialic acid cannot be accounted for by the short fiber being more abundant in the HAdV-52 virion. We also found by flow cytometry analysis that A549 cells expressed higher levels of CAR compared with another epithelial cell line (human corneal epithelial cells; HCE) (**Fig. 4B**), suggesting that the modest function of CAR during HAdV-52 binding to A549 cells was not due to low expression levels on these cells. Homology alignment of the long and short fiber knob sequences with corresponding sequences of sialic acid-interacting HAdV-37 (ocular tropism) and CAR-interacting HAdV-5 (respiratory tropism) and HAdV-12 (respiratory and intestinal tropism) revealed that, while the majority of the known CAR-interacting residues [15,40] are conserved in 52LFK, only a few of these residues are conserved in 52SFK (**S4 Fig.**). Furthermore, when examining the potential for interactions with sialic acid based on the structure of the HAdV-37 knob bound to sialic acid [41], only two out of the seven sialic acid-contacting residues are conserved in 52SFK and none of these are conserved in 52LFK. Flow cytometry analysis confirmed that 52LFK can only bind to CAR-expressing cells (**Fig. 5A**). 52SFK bound with similar efficiency to all cells (including CAR-expressing cells) but not to sialic acid-deficient Lec2 cells (**Fig. 5B**). Neuraminidase treatment of A549 cells reduced binding of 52SFK to A549 cells but not of 52LFK (**Fig. 5C**), confirming that 52SFK binds to sialic acid-containing receptors on human target cells. ELISA experiments showed that 52SFK (in solution) bound efficiently to sialylated fetuin glycoprotein (immobilized) but not to two desialylated variants of fetuin (**Fig. 5D**), this was also confirmed with surface plasmon resonance (SPR) where fetuin bound to immobilized 52SFK with an affinity of 37 μM, while for the desialylated fetuin type II a K_D_ could not be determined (**S5 Fig.)**. 52LFK did not bind to any of these proteins. SPR analysis demonstrated that the 52LFK:CAR-D1D2 (full length extracellular domain) and 52LFK:CAR-D1 interactions were of high affinity (5 and 2.6 nM, respectively; **Figs. 5E and S6**), which is in the same range as of other CAR:HAdV-knob interactions [42]. According to SPR analysis, 52SFK did not interact with CAR at all (**S7 Fig.**). As the sialic acid-containing glycan(s) used by 52SFK for binding to A549 cells are not known, we can only speculate that such monovalent interactions would probably be of low affinities, as most other protein:glycan interactions, and thereby lower than the affinity of the LFK:CAR interaction. We conclude from these results that the HAdV-52 long fiber binds to CAR and that the short fiber binds to sialic acid-containing glycans. It has been shown that cells infected with HAdV-2 (species HAdV-C) secrete an excess of fibers that unlocks junctional, intercellular CAR-CAR homodimers, resulting in increased extracellular space and improved intercellular transport of subsequently released virions [43], and similar effects have been shown for HAdV-3 (species B) penton dodecahedra [44]. It is therefore tempting to speculate that a possible function of the HAdV-52 short fiber is to mediate virion attachment to non-infected cells whereas excess of long fibers are secreted from infected cells and facilitate transmission of subsequently released virions within a tissue, or between tissues. Further support for this hypothesis is provided in that sialic acid-containing, *O*-linked glycans are abundant on the apical side of polarized epithelial cells *in vivo*, whereas CAR is mainly expressed laterally and basolaterally [45]. Thus it is plausible that virions approaching non-infected cells from the apical side have access to sialylated glycans, but not to CAR.

**Fig 6:**
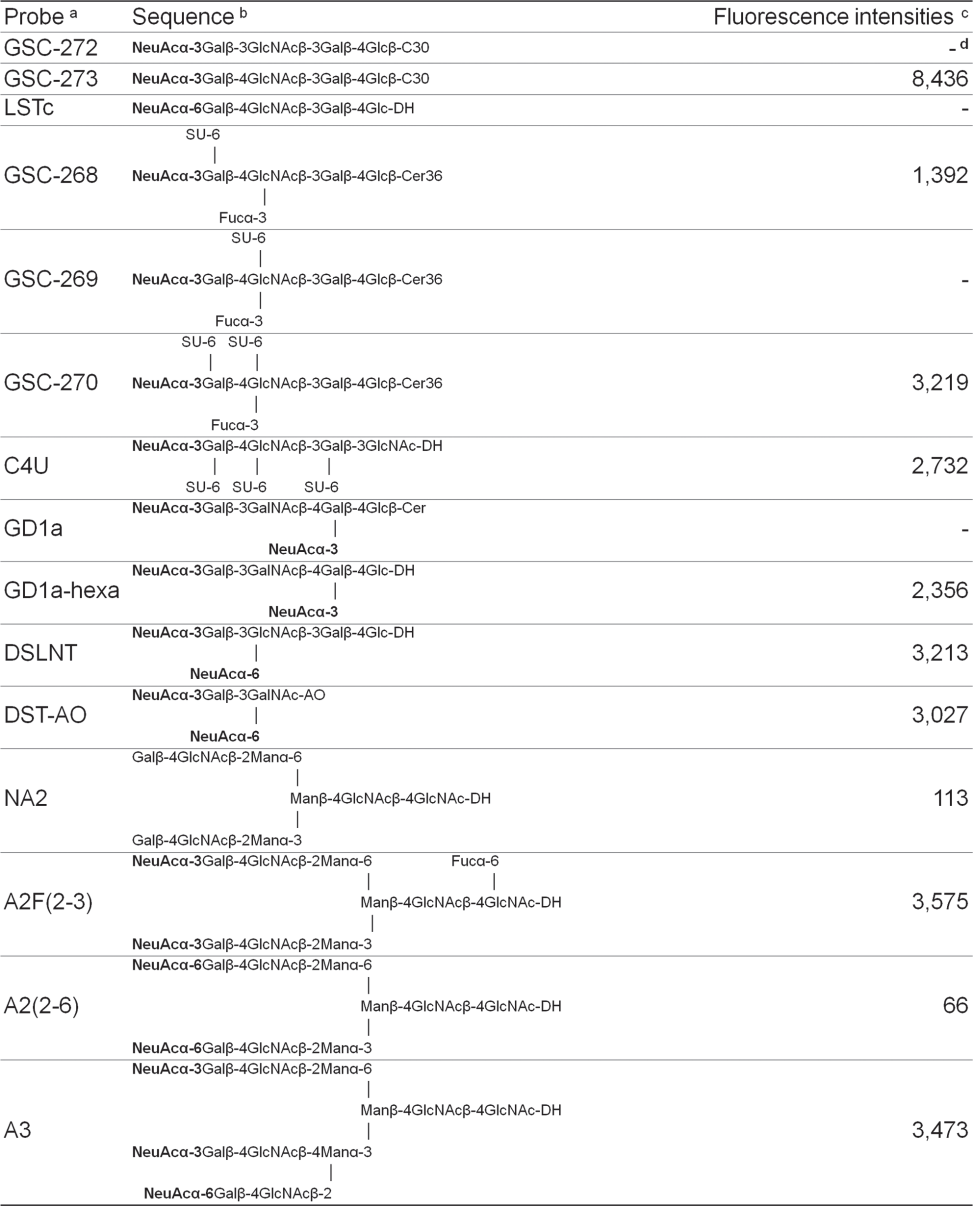
Features of 52SFK binding to selected sialyl sequences in the microarray. ^a^ The oligosaccharide probes are all lipid-linked, neoglycolipids (NGLs) or glycosylceramides and are from the collection assembled in the course of research in the Glycosciences Laboratory. ^b^ The selected α2-3-linked and α2-6-linked sialyl sequences are marked in **bold**. For definition of the lipid moieties of the probes, please see https://glycosciences.med.ic.ac.uk/docs/lipids.pdf ^c^ Numerical scores for the binding signals are shown as means of duplicate spots at 5 fmol per spot. ^d^-, signal less than 1.

HAdV-37 has been shown to interact primarily with sialic acids linked via α2,3-glycosidic bonds to galactose (Siaα2,3Gal). We found here that Siaα2,3Gal-binding *M*. *amurensis* type II (MAL-II) lectins and/or Siaα2,6Gal-binding *S*. *nigra* (SNA) lectins did not compete with HAdV-52 virion binding to A549 cells (**S8 Fig.**). We noted that α2,3-specific neuraminidase inhibited HAdV-52 virion binding to A549 cells (**Fig. 5F**), but only at 100-fold higher concentrations than what has been observed for inhibition of HAdV-37 virion binding [11]. Pretreatment of A549 cells with neuraminidase from *V*. *cholerae*, which cleaves α2,3/6/8-linked sialic acids with similar efficiencies, inhibited HAdV-52 binding at much lower concentrations. By means of glycan microarray screening we identified a number of α2,3-sialylated probes that are bound by 52SFK, whereas no binding was detected with probes that contain exclusively α2,6-sialyl linkage (**Fig. 6**). The probe most strongly bound was a synthetic glycolipid with type II (Galβ-4GlcNAc) backbone sequence (GSC-273). In contrast, no binding was detected to the type I (Galβ-3GlcNAc) analog (GSC-272). Weaker binding was observed to three of the four sulfated sialyl analogs with or without 3-linked fucose. There was also weak binding to a neoglycolipid derived from GD1a glycan, the previously described ligand for HAdV-37 [35]. No binding was detected to GD1a glycosylceramide. It should be mentioned that, although five of the ligand-positive sialyl probes in the array were glycolipids, their glycan sequences are common to glycoproteins. Among the *N*-glycan probes analysed, there was binding to probes with α2,3-linked terminal sialic acids. Collectively, we cannot exclude α2,3-linked sialic acid-containing *N*-glycans from contributing to HAdV-52 binding to A549 cells, but it is likely that other types of sialic acid-containing glycans also contribute. Our results suggest that the short fiber is capable of binding to sialic acids on *O*-glycosylated proteins on A549 cells, but that on other cell types binding to sialyl-*N*-glycans may also occur.

### Structure of the complex of HAdV-52 SFK bound to sialic acid

**Fig 7:**
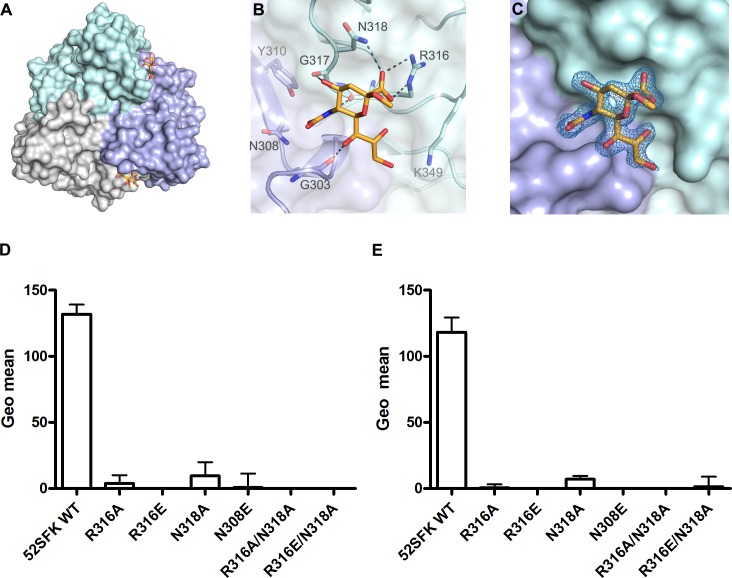
HAdV-52 interaction with sialic acid. (A) Surface representation of the HAdV-52 short fiber knob structure viewed from the top along the three-fold symmetry axis. The three monomers are shown in green, blue and grey. The 2-*O*-methyl sialic acid bound to two of three binding sites is shown as orange and red stick model. (B) Detailed view of the interactions in the ligand binding site. The side chain of R316 forms a bidentate salt bridge with the carboxyl group of sialic acid. Residues G303, R316 and G317 form backbone hydrogen bonds with the glycerol, amide and O4 groups of the sialic acid, respectively, while the side chain of N318 engages in a hydrogen bond with the sialic acid carboxylate. The methyl group is not involved in binding contacts. (C) Simulated annealing Fo-Fc omit map for the sialic acid. The map was calculated at 3.0 σ and is displayed with a radius of 1.7 Å around the ligand. 52SFK WT (wild type) and mutant binding to Pro5 (D) and A549 (E) cells. All experiments were performed three times with duplicate samples in each experiment. Error bars represent mean ± SD. * P of < 0.05, ** P of < 0.01 and *** P of < 0.001 versus control.

**Fig 8:**
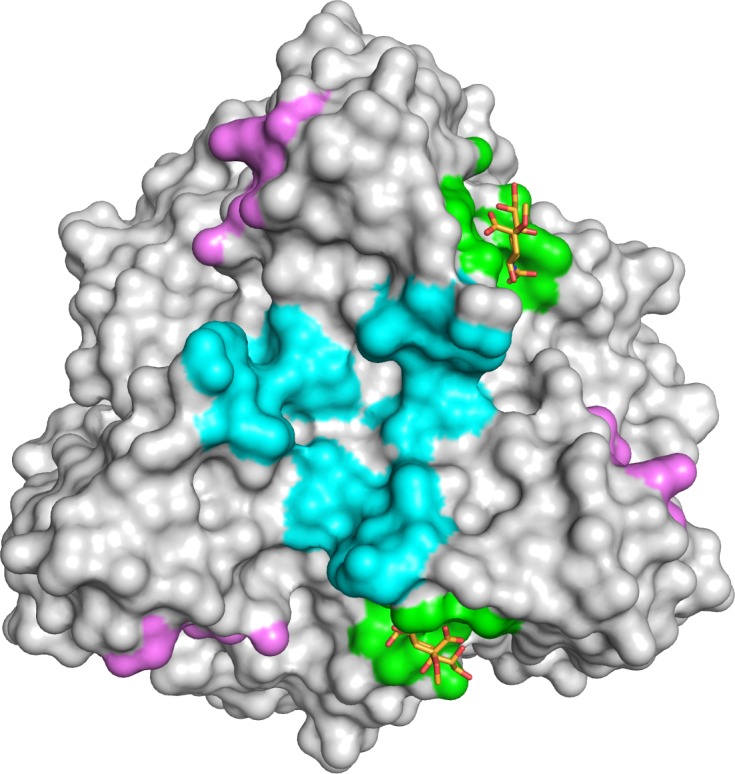
HAdV-52 has a unique sialic acid binding site compared to other sialic acid binding HAdVs. Comparison of the sialic acid binding site in HAdV-52 (green with orange sticks) with those of HAdV-37 (cyan) and CAdV-2 (magenta). The protein chains were superposed using the PyMOL (The PyMOL Molecular Graphics System, Version 1.5.0.4 Schrödinger, LLC) align tool, and the contact surface of each ligand was mapped onto HAdV-52 (calculated 4.5 Å around sialic acid).

In order to define the interactions of the HAdV-52 fiber with sialic acid, we solved the crystal structure of 52SFK in complex with 2-*O*-methyl-sialic acid (a stereochemically uniform analogue of sialic acid) at a resolution of 1.65 Å. Similar to all other known AdV fiber knob structures [13,46–48] the 52SFK has a nine-stranded antiparallel β-sandwich fold and forms a stable trimer in solution. The three shallow sialic acid binding sites of 52SFK are formed at the contact site of two neighboring monomers by the EG and GH loops at the side of the short fiber knob domain (**Fig. 7A-C**). Two of the three binding sites are partially blocked by crystal contacts, and therefore the structure contains only one fully occupied sialic acid, while a second sialic acid is visible with partial occupancy in one of the two partially blocked binding sites. The location of this binding site is distinct from those of the other structurally characterized sialic acid-binding fiber knobs, HAdV-37 and canine adenovirus type 2 (CAdV-2; included here since it is the only known sialic acid-interacting AdV besides those of species HAdV-D) (**Fig. 8**; PDB_IDs 1UXA and 2WBV). The bound 2-*O*-methyl-sialic acid is well defined by electron density (**Fig. 7C**) and engages the 52SFK mainly through contacts between the sugar’s carboxylate group and the side chains of R316 and N318 (**Fig. 7B**). The bidentate salt bridge formed by R316 is a prominent binding motif among glycan binding viruses [49,50] (**S9 Fig.**). In addition, the backbone carbonyl oxygens of R316, G317, and G303 form hydrogen bonds with the sialic acid O4, N-acetyl and glycerol-like functions, respectively (**Fig. 7B**). The tripeptide R316-G317-N318 located on the GH loop forms a hook-shaped motif (RGN motif) that is contributing most of the interactions, and that therefore largely defines the specificity of SFK52 for sialic acid. The pattern of polar contacts formed by this motif is highly similar to HAdV-37 (**S9 Fig.**) [50]. Mutating either R316 or N318 to alanine, replacing the R316 side chain with a negatively charged glutamate, or introducing a steric clash (and a polar clash, introducing a charge) at position 308 (N308E) all abolished the attachment of 52SFK to Pro-5 and A549 cells (**Fig. 7D,E**). The sugar’s O2 function, to which additional sugars would be attached in a glycan chain, is pointing away from the protein and towards the tip of the knob, suggesting that more complex glycan receptors that bind the knob with their sialic acid caps would have to face towards the capsid in order to be bound by the virus. The methyl group attached to this oxygen in the compound used for structural analysis (2-*O*-methyl-sialic acid) does not participate in interactions with the knob. Alignment of multiple knob sequences suggests that the RGN motif is conserved in the knob domain of the short fibers of other members of species HAdV-G: simian AdV-1 and -7 (**S10 Fig.**), and we therefore predict that the ability to engage sialylated receptors is shared by these HAdVs. The RGN motif is not conserved in any other known human and non-human AdV knob sequences, including the short fiber knobs of HAdV-40 and -41.

In conclusion, we have identified two types of cellular receptors used by HAdV-52, the only human member of species HAdV-G. By analogy with HAdV-40 and -41, we identified CAR as a receptor for the HAdV-52 long fiber. We also present evidence that *O*-glycosylated proteins carrying sialic acid-containing glycans serve as receptors on A549 cells for HAdV-52 short fibers. The 52LFK:CAR interaction is probably of higher affinity than the 52SFK:sialic acid interaction, however the relative importance of cellular receptors is not only determined by the affinity but also to a high extent on the abundance of the receptors. It has been shown for example by us and others that HAdV-37 binds with lower affinity to CAR (20 nM) than other HAdVs, but with even lower affinity to sialic acid-containing glycans (19 μM) [41,51]. Still, HAdV-37 uses sialic acid-containing glycoproteins as the main receptor [11,52]. Accessibility may also influence receptor usage. *In vivo*, CAR localizes to the lateral and basolateral side of polarized epithelial cells, which are not easily accessible for HAdVs, while sialic acid is abundant on the apical surface and may therefore be more available for interaction. Thus we suggest that sialylated proteins rather than CAR function as primary receptors for HAdV-52 virions on A549 cells. HAdV-52 might have retained the ability to bind to CAR as secretion of CAR-interacting fibers can disrupt CAR-CAR homodimers in the tight junctions and thereby facilitate virion escape and transmission within a tissue, or between tissues. The mode of interaction between 52SFK and its sialylated receptors is fundamentally different, both in location on the protein and in contacts formed to the ligand, from the known interactions between HAdV-37 fiber knob and sialic acid. As the sialic acid-binding RGN motif of 52SFK is not conserved in any other HAdV fiber, it appears that HAdV-52 and other members of species HAdV-G employ a unique strategy for engaging sialic acid. This is the first information presented about the receptors used by viruses in species HAdV-G, and the first describing a receptor recognized by an AdV short fiber. These findings shed light on AdV biology and tropism and may be useful for development of vectors based on members of species HAdV-G.

## Materials and Methods

### Cells, viruses and antibodies

A549 cells (gift from Dr. Alistair Kidd) were grown in Dulbecco´s modified Eagle medium (Sigma-Aldrich) supplemented with 5% fetal bovine serum (FBS: Invitrogen), 20 mM HEPES (Sigma-Aldrich) and 20 U/ml penicillin + 20 μg/ml streptomycin (Invitrogen), human corneal epithelial (HCE) cells (gift from Dr. Araki-Sasaki) were grown as previously described [53]. Pro-5 and Lec2 cells [54,55] (both purchased from LGC Promochem), Chinese hamster ovary (CHO)-CAR, CHO-MOCK (gift from Dr. Jeffrey Bergelson) [4], and CHO-CD46 (isoform BC1; gift from Dr John P. Atkinson) [56] were grown as described.

Species G HAdV-52 (strain TB3-2243)[3] and species C HAdV-5 (Ad75; source ATCC) virions were produced with or without ^35^S-labeling in A549 cells as described previously [57], with the exception that the virions were eluted in sterile phosphate buffered saline (PBS) when desalting on a NAP column (GE Healthcare). Serotype-specific rabbit polyclonal antisera to each HAdV was a gift from Dr Göran Wadell [58]. Antiserum produced against HAdV-41 virions was used for detection of HAdV-52 antigens in infection experiments.

### Virion binding experiments

Cells were detached with PBS containing 0.05% EDTA, reactivated in growth medium for one hour at 37°C (in solution), pelleted in 96 well plates (2x10^5^ cells/well) and washed with binding buffer (BB: Dulbecco´s modified Eagle medium supplemented with 20 mM HEPES, 20 U/ml penicillin + 20°g/ml streptomycin and 1% bovine serum albumin).^35^S-labeled virions (2x10^9^ virions diluted in BB, 100 μl/sample) were added to the cells and incubated for 1 h on ice. Unbound virions were washed away with BB and the cell associated radioactivity was measured in a Wallac 1409 liquid scintillation counter (Perkin-Elmer). This experiment was performed with the following additions/variations:

- i) Cells were pretreated with increasing concentrations of *Vibrio cholerae* neuraminidase (Sigma-Aldrich) or α2,3-sialidase (TaKaRa Bio Inc) for 1 h at 37°C, or, with a 1:100 dilution of anti-CAR antibody (clone RmcB, Upstate, Millipore) or with 50 μg/ml *M*. *Aamurensis* type II (MAL-II) or *S*. *Nigra* (SNA) lectins (Vector Laboratories) for 1 h on ice before addition of virions. The effect of *V*. *cholerae* neuraminidase treatment of CHO cells was examined by flow cytometry. Cells were incubated with 1μg/ml of biotinylated MAL-II lectin for 30 min on ice, followed by a 30 min incubation with a 1:100 diluted streptavidin-FITC (on ice).
- ii) Virions were preincubated for 1 h on ice with a) 100 mM sialic acid (or concentrations as indicated; N-acetylneuraminic acid, Dextra Laboratories), b) CAR-D1 (a kind gift from Dr. Paul Freimuth), c) physiological concentrations of coagulation factor IX (FIX 5 μg/ml, resulting in a 1 virion:2700 FIX ratio, Calbiochem) or coagulation factor X (FX 10 μg/ml, resulting in a 1 virion:5100 FX ratio, Haematologic Technologies Inc.) before incubation with A549 cells.
- iii) To affect both CAR and sialic acid binding, A) virions were preincubated with a combination of sialic acid and CAR-D1 1h on ice before addition to A549 cells, B) cells were preincubated with a 1:100 dilution of anti-CAR antibody on ice, and virions were preincubated with sialic acid, C) cells were pretreated with *Vibrio cholerae* neuraminidase for 1 h at 37°C and virions were preincubated with CAR-D1 for 1 h on ice before they were mixed together, or D) cells were pretreated with *Vibrio cholerae* neuraminidase for 1 h at 37°C followed by incubation with a 1:100 dilution of anti-CAR antibody on ice before addition of virions. HAdV-5 was used as a CAR-binding control, to check the efficiency of soluble CAR-D1 and anti-CAR mab.iv) Cells were pretreated with or without 2.5 μM P4 [(1*R*,2*R*)-1-phenyl-2-hexadecanoylamino-3-pyrrolidino-1-propanol] or 2.5 μM inactive enantiomer of P4 (1*S*,2*S*, both kindly provided by Dr Roland L. Schnaar) for 5 days at 37°C to inhibit glycolipid biosynthesis via the glycosylceramide synthase enzyme [59]. Media was changed after 3 days when new P4 was added. The effect of P4 on the cells was analyzed by performing flow cytometry using ganglioside G_M1_-binding AF488-conjugated cholera toxin subunit B (Invitrogen, Molecular probes). v) Cells were pretreated with 0.3 μg/ml tunicamycin (Sigma Aldrich) for 24 h at 37°C to inhibit *N-*glycosylation, or with 3 mM benzyl-α-GalNAc (Sigma-Aldrich) for 48 h at 37°C to inhibit *O-*glycosylation. To determine the effect of tunicamycin, cells were incubated with FITC-conjugated Phaseolus vulgaris erythroagglutinating lectin (PHA-E; Vector Laboratories) for 1 h on ice, washed and analyzed with flow cytometry. Cell viability was verified with trypan blue staining prior to binding experiments

### Infection experiments

Pro-5, Lec2, or A549 cells, grown as monolayers on glass slides in 24-well plates, were washed three times with serum-free medium and treated with or without 10 mU/well of *Vibrio cholerae* neuraminidase for 1 h at 37°C. Virions were added to the cells and incubated for 1 h on ice. After incubation, the wells were washed three times with serum-free medium in order to remove unbound virions. Cell culture medium containing 1% FBS was added and the plates were incubated for 44 h at 37°C. Thereafter the glass slides were washed with PBS (pH 7.4) once, fixed with methanol and stained with polyclonal rabbit anti-HAdV diluted 1:200 for 1 h at room temperature. The slides were washed twice with PBS and incubated for an additional hour with a FITC-conjugated swine anti-rabbit IgG antibody (DakoCytomation) diluted 1:100 in PBS. After washing, the slides were mounted and examined in a fluorescence microscope using 20 X magnification (Axioskop2, Carl Zeiss). Ten pictures was taken of each well and the number of infected cells was calculated using ImageJ [60]. In one experiment, virions were preincubated with or without physiological concentrations of FIX (5 μg/ml, equal to 1:60000 virion:FIX ratio) or FX (10 μg/ml, equal to 1:110000 virion:FX ratio) for 1 h on ice before addition to cells.

### Cloning and purification of fiber knobs

DNA isolation from HAdV-52 virions was performed by using the Blood & Cell Culture DNA Mini kit (Qiagen Nordic). DNA fragments encoding HAdV-52 long fiber knob (52LFK) and HAdV-52 short fiber knob (52SFK) were amplified by polymerase chain reaction (PCR) using KOD Hot Start (Novagen, Merck) and the following primers (DNA Technology): 52LFK forward (5´-aaaaggatccggaaacatagctgtttctcct), reverse (5´-aaaacccgggcggaggaagccttactgtgcgtgt), 52SFK forward (5´-aaaaggatccaggtttaacagcagt-ggagcc), reverse (5´-aaaacccgggagggttttattgttcggtaatgtagca). Fragments were then cloned into a pQE30Xa expression vector encoding an N-terminal His-tag (Qiagen) using restriction sites for BamHI and XmaI (Fermentas, ThermoFisher Scientific). All constructs were confirmed by sequencing (Eurofins MWG Operon). Proteins were expressed in *Escherichia coli* (strain M15) and purified with Ni-NTA agarose beads according to protocol from the supplier (Qiagen). Proteins were analyzed by denaturing gel (NuPAGE Bis-Tris, Invitrogen, Life Technologies) and western blot with monoclonal antibodies directed against the His-tag (Qiagen).

### Fiber knob mutants

Six different 52SFK mutants were created using a QuikChange mutagenesis kit (Agilent) according to their protocol. The following mutants were created: 1) R316A,2) R316E, 3) N318A, 4) N308E, 5) R316A/N318A, and 6) R316E/N318A. Correct trimerization of all proteins was confirmed with gas-phase electrophoretic mobility molecular analysis (GEMMA)[61] (**S11 Fig.,** showing 52SFK wt and one representative mutant; R316A), which is a protein oligomer measurement technique where the protein solution is converted into gas phase by a charged reduced electrospray process. The particles are separated according to size in a differential mobility analyzer and quantified by a particle counter. All mutant and wt fiber knobs were analyzed in the same manner: G-25 columns (GE Healthcare) were used for buffer exchange to 20mM ammonium acetate buffer, pH 7.8 containing 0.005% (v/v) Tween 20. Buffer exchange was done to remove the non-volatile salts from the protein solution. The concentrations for 52SFK wt and mutants 1, 2 and 3 were 0.05 mg/ml while for mutants 4, 5 and 6 it was 0.06 mg/ml. Three to five scans were taken with the GEMMA system (TSI Corp.) for each sample with a capillary pressure of either 1.7 or 3.7 psi. These parameters depended on the protein sample and the stability of the signal. Each sample was scanned for 120 seconds per scan at the size range of 2.55–255 nm. For molecular mass calculations, a particle density of 0.58 g/cm^3^ was used.

### Fiber knob binding experiments

Cells were detached with PBS-EDTA, reactivated in growth medium for one hour at 37°C, pelleted in 96 well plates (2x10^5^ cells/well) and washed once with BB. The cells were then incubated with 10 μg/ml of 52SFK or 52LFK in 100 μl BB for one hour on ice. Unbound fiber knobs were washed away with PFN (PBS containing 2% FBS and 0.01% NaN_3_) and the cells were then incubated with an anti RGS-His mouse monoclonal antibody (Qiagen; diluted 1:200 in PFN) for 30 min. Followed by one wash with PFN, the cells were incubated with polyclonal rabbit-anti-mouse FITC antibodies (Dako Cytomation; diluted 1:20 in PFN) for 30 min on ice. Thereafter the cells were washed with PFN and analyzed with flow cytometry using FACSLSRII instrument (Becton Dickinson). Results were analyzed using FACSDiva software (Becton Dickinson). This experiment was performed with the following additions/variations: The cells were i) grown in the presence or absence of benzyl-α-GalNAc (as described above), ii) preincubated with or without different concentrations of proteases (ficin, proteinase K and bromelain; all from Sigma-Aldrich) for 30 min at 37°C before incubation with fiber knobs, and iii) treated with or without *Vibrio cholerae* neuraminidase for 1 h at 37°C before incubation with fiber knobs.

### Western blot

Purified HAdV-52 virions were resolved on 10% Bis-Tris denaturing gels (NuPAGE, Invitrogen, Life Technologies) and transferred to Trans-Blot nitrocellulose membranes (Bio-Rad Laboratories, Solna, Sweden) by electroblotting. The membrane was blocked with 5% milk in PBS-T (PBS supplemented with 0.05% Tween20). Staining was carried out using 1:5000 dilution of a monoclonal anti-adenovirus fiber antibody (epitope region suggested by the manufacturer: MKRARPSEDTFNPVYPY, clone 4D2, ab3233, Abcam) in PBS-T with 2.5% milk, followed by a 1:1000 dilution of a HRP-conjugated rabbit anti-mouse IgG antibody (Dako Cytomation) in PBS-T with 2.5% milk. The fibers were then detected by chemiluminescence using super signal west pico or femto (Thermo Scientific) and visualized using the multipurpose CCD camera system FujiFilm LAS-4000. Pictures were taken every 10s and the relative abundance of the two fibers were evaluated using ImageJ.

### Cellular expression of CAR

A549 and HCE cells were detached with PBS-EDTA, reactivated in growth medium for one hour at 37°C, pelleted in 96 well plates (2x10^5^ cells/well) and washed once with PFN. The cells were then incubated with a mouse monoclonal antibody directed against CAR (E1-1, Merck Millipore) for 30 min on ice followed by one wash with PFN. A polyclonal rabbit-anti-mouse FITC antibody (Dako Cytomation) was added to the cells and incubated for 30 min on ice followed by one wash with PFN before flow cytometry analysis.

### ELISA

96-well plates (Nunc maxisorp, Thermo Scientific) were coated with 1 μg/ml of fetuin or asialofetuin type I or II (Sigma-Aldrich) for 2 h at room temperature (RT) in coating buffer (bicarbonate/carbonate coating buffer 100 mM, pH 9.6). Meanwhile fiber knobs (0.4 μg/ml) were preincubated with monoclonal anti RGS-His antibodies (Qiagen; dilution 1:1000) in PBS-T for 1 h at RT. The wells were then washed four times with PBS-T and incubated with the fiber knob mixtures for 1 h at RT. After washing, the plate was incubated with a HRP-conjugated rabbit anti-mouse IgG antibody (Dako Cytomation; diluted 1:2000 in PBS-T) for 1 h at RT. The wells were washed again and incubated with 100 μl enhanced K-Blue TMB substrate (Neogen Europe) for 15 min and the reaction was then stopped by addition of 100 μl 1 M H_2_SO_4_. The absorbance was measured at 450 nm using Tecan infinite F2000 Pro (Tecan Nordic AB).

### Determination of binding parameters by use of surface plasmon resonance (SPR)

All SPR experiments were performed at 25°C with a Biacore T100 instrument and a data collection rate of 1 Hz. For CAR interaction studies: CM5 sensor chips, amine-coupling kit, and HBS-EP+ buffer (10 mM HEPES, 150 mM NaCl, 3 mM EDTA, 0.005% [vol/vol] surfactant P20, pH 7.4) were all purchased from GE Healthcare. Recombinant human CAR (CXADR Fc chimera; R&D Systems; full length extracellular D1D2 domain), or CAR-D1 was coupled to the CM5 sensor chip by using the amine coupling reaction according to the manufacturer’s instructions, resulting in an immobilization density of 900–1100 RU. The surface of the upstream flow cell was subjected to the same coupling reaction in the absence of protein and used as reference. All binding assays were carried out at 25°C, and HBS-EP+ buffer was used as running buffer. The analytes (52LFK and 52SFK) were serially diluted in running buffer to prepare a two-fold concentration series ranging from 8 nM to 2 μM, and then injected in series over the reference and experimental biosensor surfaces for 120 s at a flow rate of 30 μl/min. Blank samples containing only running buffer were also injected under the same conditions to allow for double referencing. After each cycle, the biosensor surface was regenerated with a 60 s pulse of 10 mM Tris-Glycine pH 1.5 at a flow rate of 30 μl/min. For 52SFK interaction studies: Ni-NTA sensor chips, and HBS-EP+ buffer were purchased from GE Healthcare. 52SFK was diluted in running buffer (HBS-EP+) to a concentration of 0.03μM and captured on the Ni-NTA sensor chip according to the manufacturer’s instructions, resulting in an immobilization density of 700 RU. In short: an automated program cycle of the following sequence: (1) activation of the sensor chip with Ni(II), (2) capture of 52SFK (3) analyte injection, (4) regeneration of the surface with 0.3 M EDTA, and (5) rinse with HBS-EP+ without EDTA. All steps were performed at a flow rate of 30 μl/min. All binding assays were carried out at 25°C, and HBS-EP+ buffer was used as running buffer. The analytes (fetuin and asialofetuin type II) were serially diluted in running buffer to prepare a two-fold concentration series ranging from 125 to 1 μM, and then injected in series over the reference and experimental biosensor surfaces for 180 s and a dissociation time of 100 s. Blank samples containing only running buffer were also injected under the same conditions to allow for double referencing.

### Glycan microarray

Microarrays were composed of lipid-linked oligosaccharide probes robotically printed on nitrocellulose-coated glass slides at 2 and 5 fmol per spot in duplicate using a non-contact instrument as described previously [62]. Binding signals were probe-dose dependent. For 52SFK binding, the results of 15 oligosaccharide probes at 5 fmol per spot are shown in Fig. 6. The full microarray data will be described elsewhere. The microarray binding assay of the recombinant His-tagged 52SFK was performed at 20°C essentially as described [63]. In brief, the arrayed slide was blocked for 1 h with 5 mM HEPES pH 7.4, 150 mM NaCl, 5mM CaCl2, 0.3% (v/v) Blocker Casein (Pierce), 0.3% (w/v) bovine serum albumin (Sigma) (0.3% casein/0.3% BSA). 52SFK was pre-complexed with mouse monoclonal anti-poly-histidine (Ab1) and biotinylated anti-mouse IgG antibodies (Ab2) (both from Sigma) in a ratio of 4:2:1 (by weight). The 52SFK-antibody pre-complexes were prepared by pre-incubating Ab1 with Ab2 for 15 min at ambient temperature, followed by addition of 52SFK and incubation for an additional 15 min on ice. The VP1-antibody complexes were diluted in 0.3% casein/0.3% BSA, to give a final 52SFK concentration of 150 μg/ml, and overlaid onto the arrays at 20°C for 2 h. Binding was detected with Alexa Fluor-647-labelled streptavidin (Molecular Probes); imaging and data analysis was as described [62].

### Structural analysis of HAdV-52:glycan interactions

**Expression and purification.** Residues 183–363 of the HAdV-52 short fiber (accession # DQ923122.2) were cloned into a pQE-30Xa vector (Qiagen) for expression of SFK52 (as described above). This construct was expressed in E. coli BL-21 DE3 at 20°C for approximately 16h after induction with 0.5 mM IPTG. Cells were harvested, resuspended in pellet buffer A_His_ [50 mM Tris (pH 7.5), 250 mM NaCl, 5% (V/V) glycerol, 10 mM imidazole] supplied with PMSF [1 mM] and lysozyme [1 mg/ml], and incubated at 4°C with agitation for 30 minutes. Cells were sonicated at maximal microtip setting with four two-minute cycles with 0.5 seconds pulses at a rate of 1 Hz. The resulting solution was centrifuged at 43,000 x g at 4°C for one hour, and the supernatant was filtered through 0.45 and 0.20 μM nitrocellulose filters. HAdV-52 was loaded onto a HisTrap Nickel IMAC column (GE Healthcare) using the Äkta Prime FPLC system and washed with buffer A_His_ until the UV absorbance returned to baseline. After a washing step with a 10% solution of B_His_ [20 mM Tris (pH 7.5), 250 mM NaCl, 5% glycerol, 500 mM imidazole] the protein was eluted by a gradient of 50–500 mM imidazole (10–100% B_His_). 20 mM DTT was added to the samples, followed by concentration and purification on a Superdex HiLoad 16/60 SD200 column (GE Life Sciences). During this step, the buffer was changed to gel filtration buffer [30 mM Tris (pH 7.5), 150 mM NaCl]. Purity was checked by SDS-PAGE analysis. Prior to crystallization, protein was concentrated to 8.5 mg/mL using a Millipore concentrator.

**Crystallization, soaking and structure determination**. For the high resolution structure, crystals of SFK52 were grown by hanging drop vapor diffusion in a reservoir solution of 15% (w/V) PEG_1000_, 12.5% (w/V) PEG_3350_, 12.5% (w/V) MPD, 20 μM of each Na L-glutamate, DL-alanine, glycine, DL-lysine HCl, and DL-serine, 0.1 M Tris/Bicine (pH = 8.5) using a combination of micro-and macroseeding. No cryoprotection was necessary. For complex formation, crystals were soaked with mother liquor supplied with 20 mM 2*-O-*methyl sialic acid for 1 hour. Data were collected at the PXIII beam line (SLS Villigen, Switzerland) at a wavelength of 1.000 Å using a Pilatus 2M detector. Initial phases were obtained by molecular replacement with Phaser [64] using a CHAINSAW model derived from the HAdV-12 fiber knob structure (PDB ID: 1NOB). Refinement was carried out using Phenix [65] and Buster [66] using threefold NCS averaging, TLS ans isotropic B factor refinement. Data statistics are given in **S12 Fig**. Structural figures were prepared with PyMOL (The PyMOL Molecular Graphics System, Version 1.5.0.4 Schrödinger, LLC).

### Alignment

Homology sequence alignment was performed using the Clustal W algorithm and the online server: http://www.ebi.ac.uk/Tools/clustalw2/index.html

### Statistical analysis

All experiments were performed three times with duplicate samples in each experiment. The results are expressed as means ± standard deviations and either t-test or one-way ANOVA with Dunnett's post test was performed using GraphPad Prism version 4.00 for Windows, GraphPad Software, San Diego California USA. P-values < 0.05 were considered statistically significant.

## Supporting Information

S1 Fig MAL-II binding to CHO-cells. Binding of the sialic acid binding MAL-II lectin to CHO cells expressing or lacking known HAdV receptors. Pro-5 is a sialic acid-positive, reference cell line, CHO-CD46 and CHO-CAR cells express human CD46 and CAR, respectively. CHO-MOCK is mock transfected with respect to CHO-CAR. Black bars show MAL-II binding to cells after pretreatment with *V*. *cholerae* neuraminidase.
(TIF)

S2 Fig Effect of RmcB and soluble CAR-D1 on HAdV-5 binding to A549 cells. ^35^S-labeled HAdV-5 virion binding to A549 cells after virion preincubation with or without soluble CAR-D1 or cell preincubation with or without mouse anti-CAR mab (clone RmcB). The experiment was performed three times with duplicate samples in each experiment. Error bars represent mean ± SD. ** P of < 0.01.
(TIF)

S3 Fig Control of P4 active/inactive and tunicamycin function. (A) Ganglioside G_M1_-binding AF488-conjugated cholera toxin subunit B (CT-B) binding to A549 cells pretreated with active or inactive forms of P4 (inhibitor of glycolipid synthesis). CT-B binding was analyzed using flow cytometry. (B) *N*-linked glycan-binding Phaseolus vulgaris erythroagglutinating lectin (PHA-E; FITC-conjugated) binding to A549 cells pretreated with tunicamycin (inhibitor of *N*-linked glycan synthesis. Note that PHA-E can only bind to a specific subset of N-linked glycans). PHA-E binding was analyzed using flow cytometry. All experiments were performed three times with duplicate samples in each experiment. Error bars represent mean ± SD. * P of < 0.05, ** P of < 0.01 and *** P of < 0.001 versus control.
(TIF)

S4 Fig Potential CAR- and sialic acid-interacting residues in AdV fiber knobs. CAR-engaging residues in the knobs of HAdV-5[40] and -12[15] are shown on yellow background. Residues in direct contact with CAR are Asp191, Leu202, Lys205 (HAdV-12) and in indirect contact (via water) are Pro193, Pro194, Val226, Lys227, Gln263, Gln270, Ser273, Val274, Asn296, and Glu299 (HAdV-12). CAR-interacting residues of HAdV-5 (identified by mutagenesis): Ser193, Pro194, Lys201, Lys205, Asp259, Pro260, Glu261, Tyr262 and Tyr276. Sialic acid-engaging residues in the knob of HAdV-37 [41,50] and CAdV-2 [51] are shown on blue background. Residues in direct contact with sialic acid are Tyr312, Pro317, and Lys345 (all HAdV-37) and Ser237, Gln238, Ser240, Asn256 and Arg336 (all CAdV-2) and residues in indirect contact are Tyr 308, Thr310, Val322 and Ser344 (all HAdV-37). Potentially conserved CAR- and sialic acid-interacting residues in 52LFK and 52SFK are shown on yellow and blue backgrounds, respectively. Secondary-structure beta strands elements of HAdV-5 are indicated with arrows.
(TIF)

S5 Fig Surface plasmon resonance analysis of fetuin and asialofetuin type II (in solution) binding to 52SFK (immobilized). A twofold dilution series of fetuin and asialofetuin is shown, ranging from 125 μM to 1 μM. The affinity of the 52SFK:fetuin interaction was calculated to 37 μM, whereas no affinity could be calculated for the 52SFK:asialofetuin interaction. Results are shown as response units (RU).
(TIF)

S6 Fig Affinity and kinetics of HAdV-52 long fiber interaction with CAR-D1. Surface plasmon resonance analysis of 52LFK (in solution) binding to CAR-D1 (immobilized). A twofold dilution series of 52LFK is shown, ranging from 2 μM to 8 nM. Results are shown as response units (RU).
(TIF)

S7 Fig Surface plasmon resonance analysis of 52SFK (in solution) binding to CAR-D1D2 (immobilized). No affinity could be calculated for the HAdV-52 short fiber knob interaction with CAR-D1D2. Results are shown as response units (RU). The experiment was performed three times and the figure shows one representative set of results.
(TIF)

S8 Fig HAdV-52 binding is not affected by preincubation of cells with MAL-II and or SNA lectins. ^35^S-labeled HAdV-52 virion binding to A549 cells preincubated with *M*. *Amurensis* lectin type II (MAL-II; preferentially binds to α2,3-linked sialic acid), *S*. *Nigra* lectin (SNA; preferentially binds to α2,6-linked sialic acid), or both. Virion binding was quantified by liquid scintillation counting. The experiment was performed three times with duplicate samples in each experiment. Error bars represent mean ± SD.
(TIF)

S9 Fig Comparison of the polar contacts of HAdV-52 and HAdV-37 bound to sialic acid. The sialic acid moieties of HAdV-52 and HAdV-37[50] (PDB-ID: 1UXA) were superimposed using the “align” function in PyMOL (The PyMOL Molecular Graphics System, Version 1.5.0.4 Schrödinger, LLC). The sialic acid moiety of HAdV-52 is shown in orange, and the sialic acid bound to HAdV-37 is overlaid as a ghost. Polar contacts formed with HAdV-52 and the respective residues are colored orange, contacting residues of HAdV-37 and the respective bonds are colored light blue. Although the binding pocket of HAdV-37 is located in an entirely different part of the knob and the interacting amino acids are not conserved, the polar contacts formed are highly similar to those of the RGN motif. The salt bridge contributed by R316 in HAdV-52 is formed by K345 in HAdV-37. The hydrogen bonds formed with the sugar’s O4 and N-acetyl group are also retained.
(TIF)

S10 Fig Conservation of sialic acid-interacting residues in 52SFK. Sialic acid-interacting residues in 52SFK are shown on red background, together with similar, potential sialic acid-interacting residues of other AdV:s. Representative types have been selected from human species A-G and canine species A (HA-G and CA, respectively). Secondary-structure elements (beta strands) of HAdV-5 are indicated by arrows.
(TIF)

S11 Fig Trimerization of HAdV-52 knobs. A) 52SFK wt and B) 52SFK R316A mutant were separated according to size using gas-phase electrophoretic mobility molecular analysis (GEMMA). Results are shown as number of molecules (particles) in respect to molecular weight (kDa).
(TIF)

S12 Fig X-ray data collection and refinement statistics. Values for the highest resolution shell are shown in parenthesis.
(TIF)
