# Supplementary figures and images for: Human Adenovirus 52 Uses Sialic Acid-containing Glycoproteins and the Coxsackie and Adenovirus Receptor for Binding to Target Cells
Source: PLoS Pathog. 2015 Feb 12;11(2):e1004657. doi: 10.1371/journal.ppat.1004657 (PMC4335501; doi:10.1371/journal.ppat.1004657)

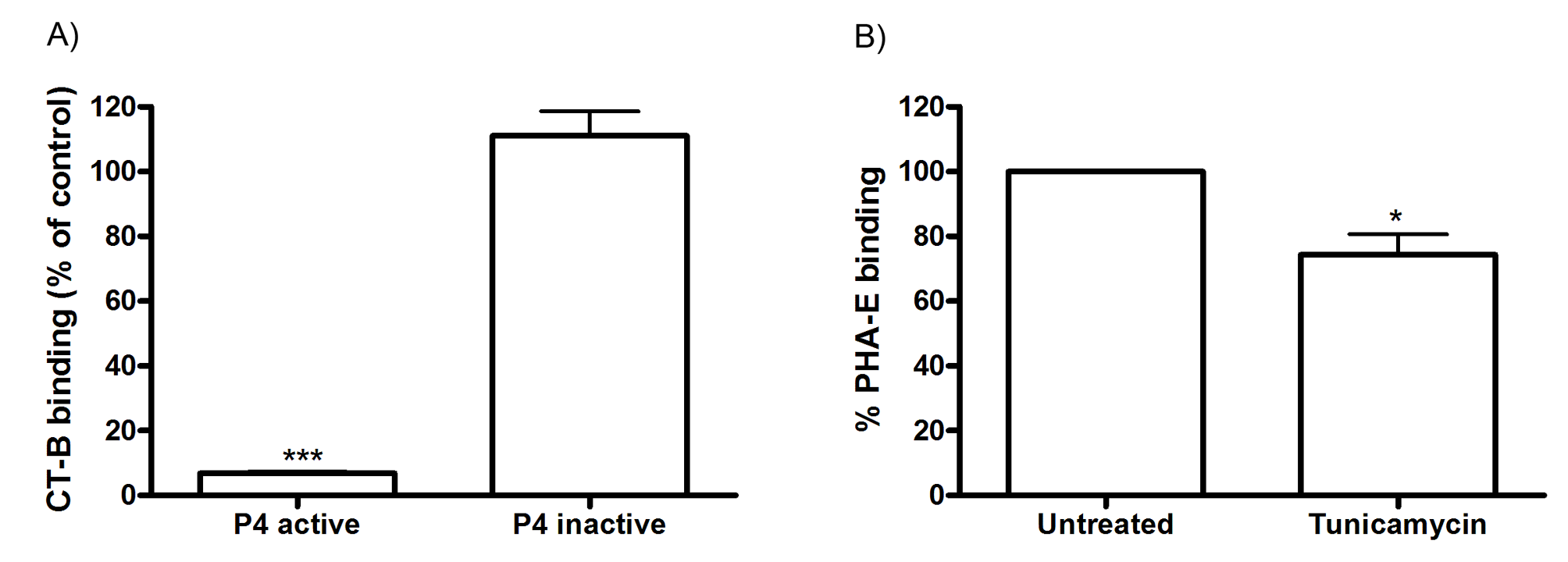

Supplement: S3 Fig — (A) Ganglioside GM1-binding AF488-conjugated cholera toxin subunit B (CT-B) binding to A549 cells pretreated with active or inactive forms of P4 (inhibitor of glycolipid synthesis). CT-B binding was analyzed using flow cytometry. (B) N-linked glycan-binding Phaseolus vulgaris erythroagglutinating lectin (PHA-E; FITC-conjugated) binding to A549 cells pretreated with tunicamycin (inhibitor of N-linked glycan synthesis. Note that PHA-E can only bind to a specific subset of N-linked glycans). PHA-E binding was analyzed using flow cytometry. All experiments were performed three times with duplicate samples in each experiment. Error bars represent mean ± SD. * P of < 0.05, ** P of < 0.01 and *** P of < 0.001 versus control. (TIF) [file ppat.1004657.s003.tif]

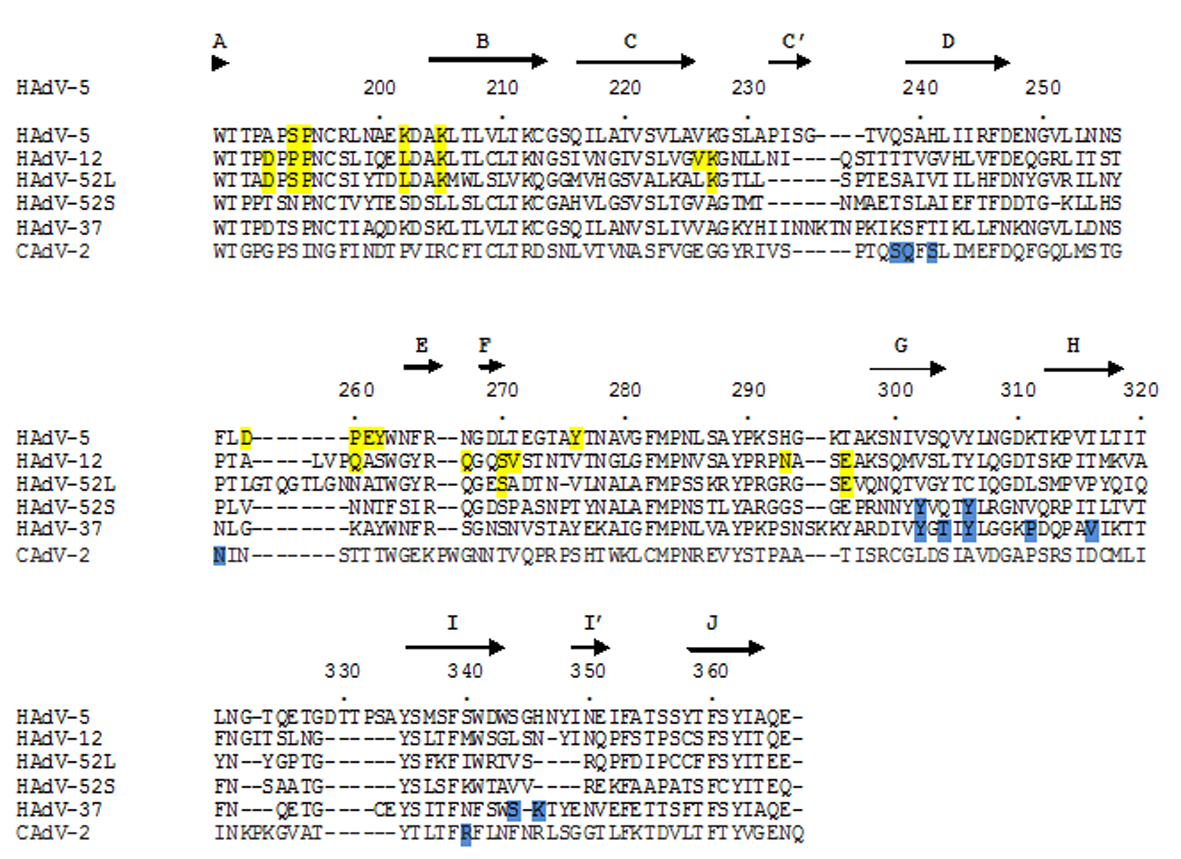

Supplement: S4 Fig — CAR-engaging residues in the knobs of HAdV-5[40] and -12[15] are shown on yellow background. Residues in direct contact with CAR are Asp191, Leu202, Lys205 (HAdV-12) and in indirect contact (via water) are Pro193, Pro194, Val226, Lys227, Gln263, Gln270, Ser273, Val274, Asn296, and Glu299 (HAdV-12). CAR-interacting residues of HAdV-5 (identified by mutagenesis): Ser193, Pro194, Lys201, Lys205, Asp259, Pro260, Glu261, Tyr262 and Tyr276. Sialic acid-engaging residues in the knob of HAdV-37 [41,50] and CAdV-2 [51] are shown on blue background. Residues in direct contact with sialic acid are Tyr312, Pro317, and Lys345 (all HAdV-37) and Ser237, Gln238, Ser240, Asn256 and Arg336 (all CAdV-2) and residues in indirect contact are Tyr 308, Thr310, Val322 and Ser344 (all HAdV-37). Potentially conserved CAR- and sialic acid-interacting residues in 52LFK and 52SFK are shown on yellow and blue backgrounds, respectively. Secondary-structure beta strands elements of HAdV-5 are indicated with arrows. (TIF) [file ppat.1004657.s004.tif]

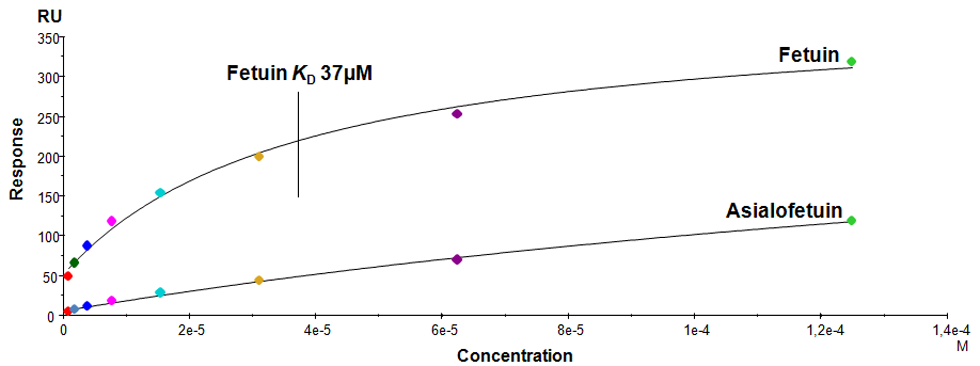

Supplement: S5 Fig — A twofold dilution series of fetuin and asialofetuin is shown, ranging from 125 μM to 1 μM. The affinity of the 52SFK:fetuin interaction was calculated to 37 μM, whereas no affinity could be calculated for the 52SFK:asialofetuin interaction. Results are shown as response units (RU). (TIF) [file ppat.1004657.s005.tif]

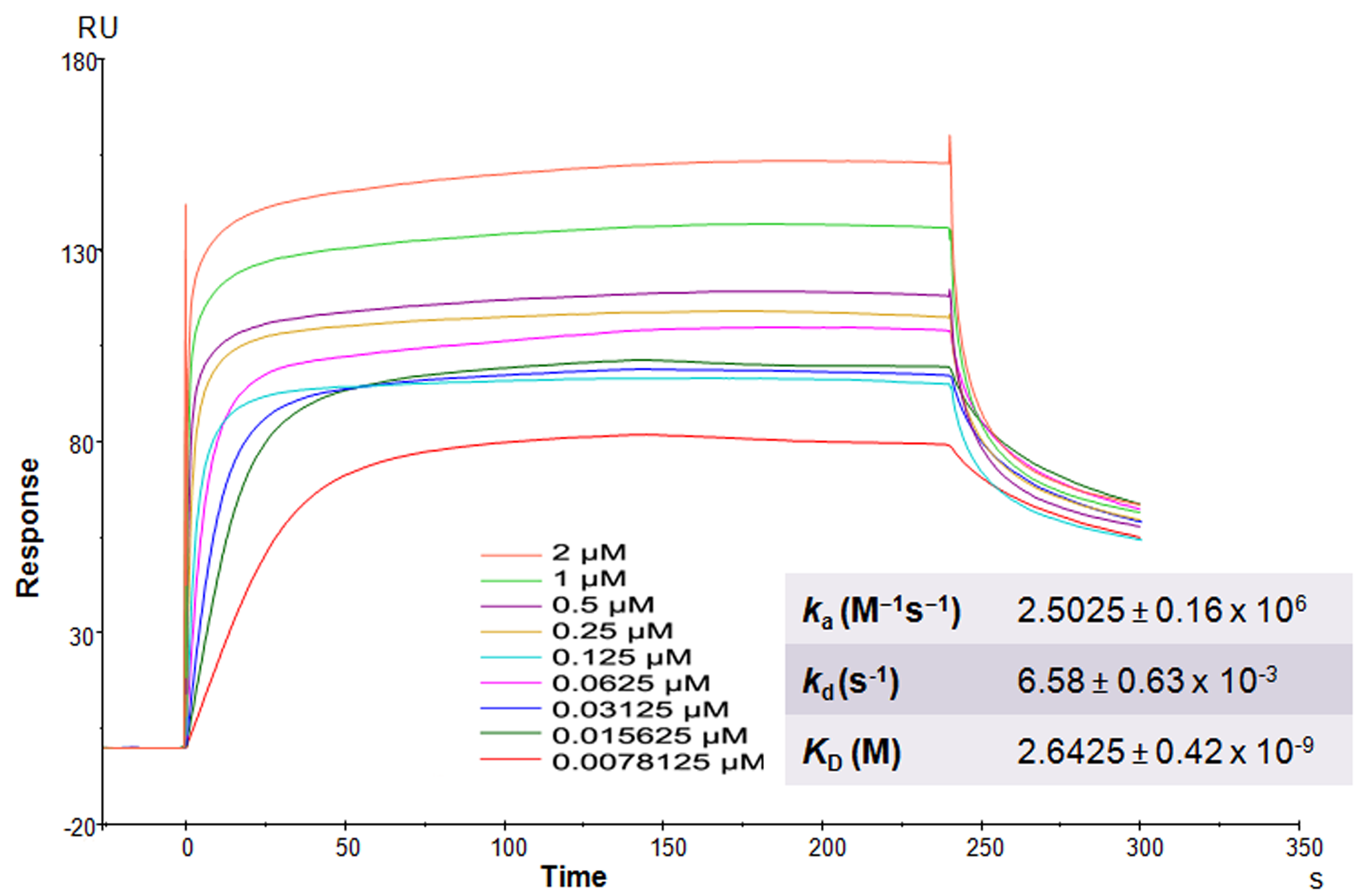

Supplement: S6 Fig — Surface plasmon resonance analysis of 52LFK (in solution) binding to CAR-D1 (immobilized). A twofold dilution series of 52LFK is shown, ranging from 2 μM to 8 nM. Results are shown as response units (RU). (TIF) [file ppat.1004657.s006.tif]

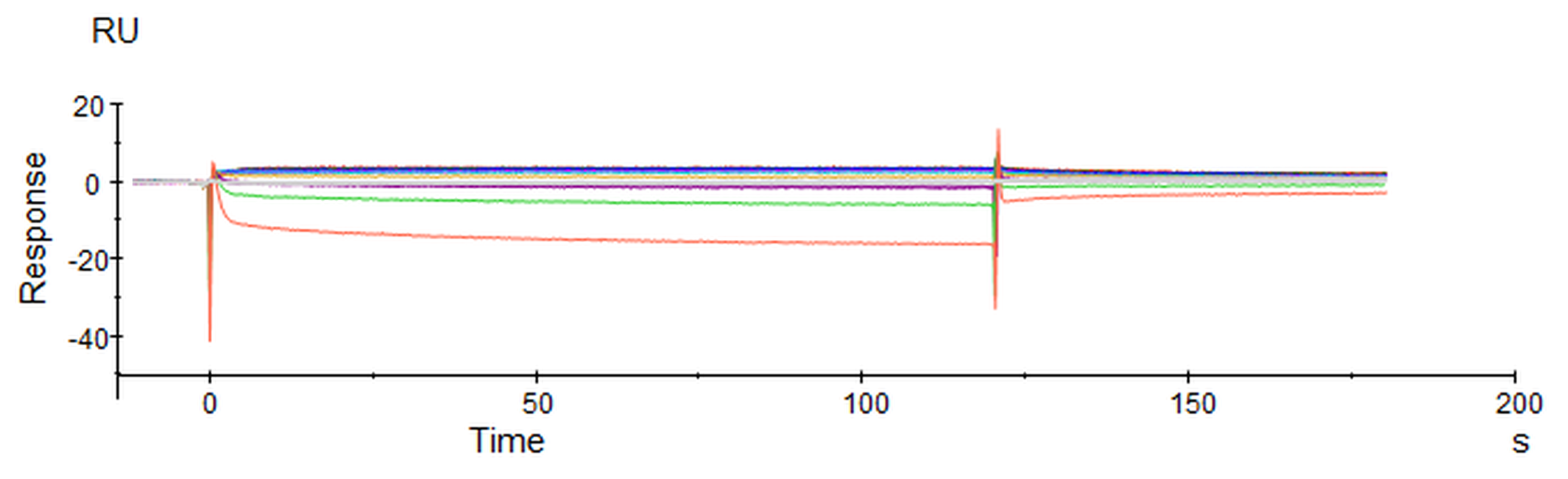

Supplement: S7 Fig — No affinity could be calculated for the HAdV-52 short fiber knob interaction with CAR-D1D2. Results are shown as response units (RU). The experiment was performed three times and the figure shows one representative set of results. (TIF) [file ppat.1004657.s007.tif]

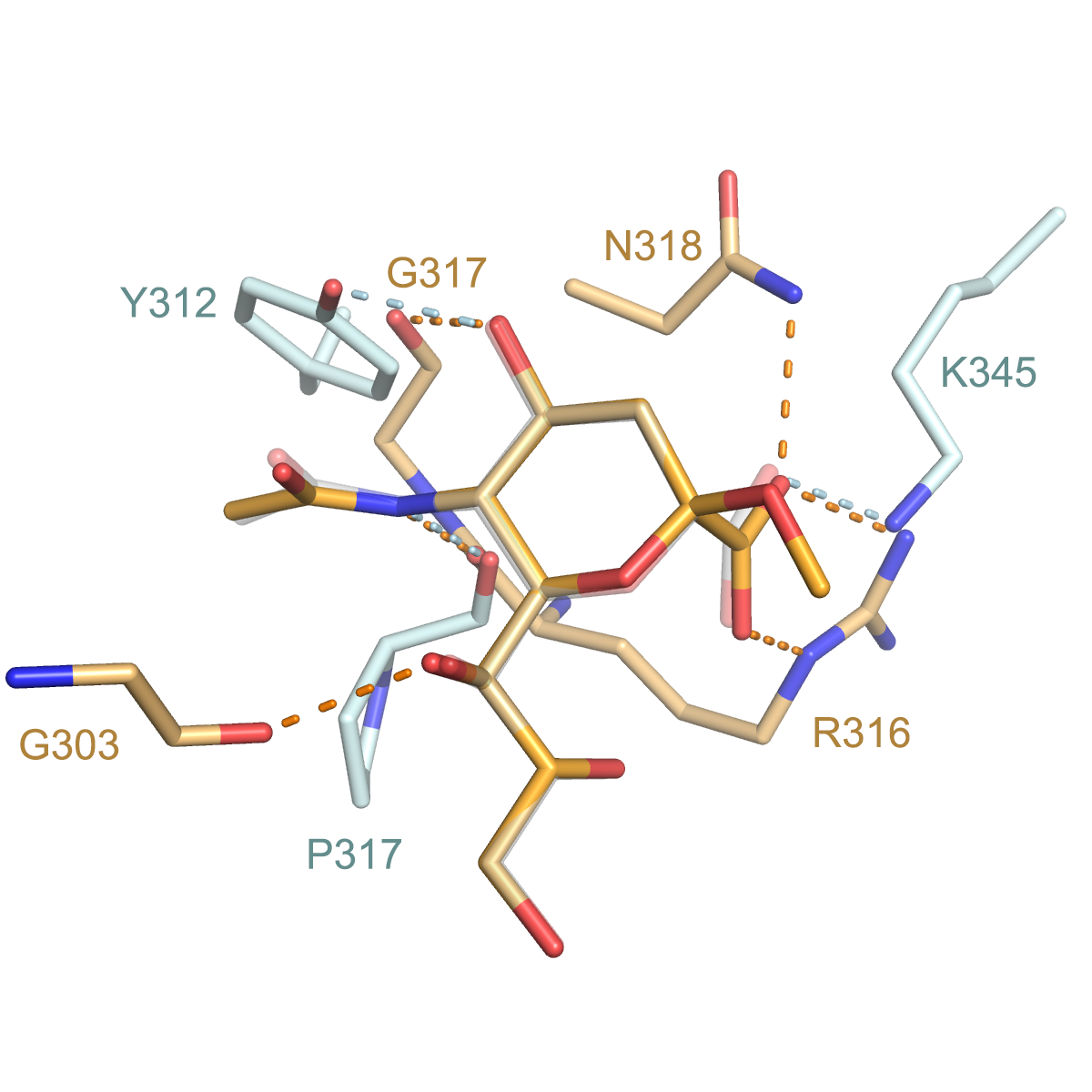

Supplement: S9 Fig — The sialic acid moieties of HAdV-52 and HAdV-37[50] (PDB-ID: 1UXA) were superimposed using the “align” function in PyMOL (The PyMOL Molecular Graphics System, Version 1.5.0.4 Schrödinger, LLC). The sialic acid moiety of HAdV-52 is shown in orange, and the sialic acid bound to HAdV-37 is overlaid as a ghost. Polar contacts formed with HAdV-52 and the respective residues are colored orange, contacting residues of HAdV-37 and the respective bonds are colored light blue. Although the binding pocket of HAdV-37 is located in an entirely different part of the knob and the interacting amino acids are not conserved, the polar contacts formed are highly similar to those of the RGN motif. The salt bridge contributed by R316 in HAdV-52 is formed by K345 in HAdV-37. The hydrogen bonds formed with the sugar’s O4 and N-acetyl group are also retained. (TIF) [file ppat.1004657.s009.tif]

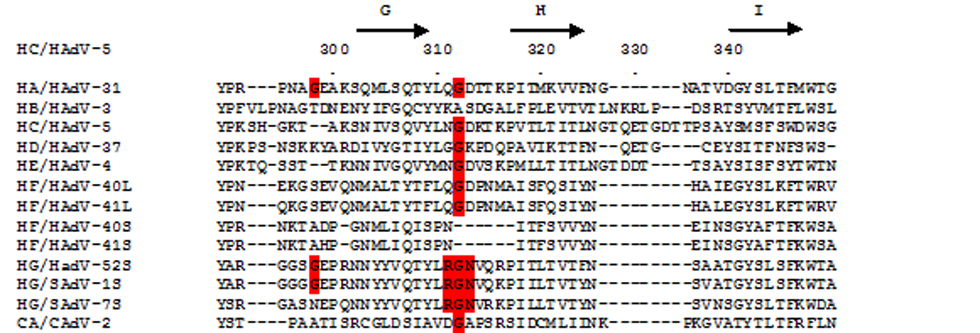

Supplement: S10 Fig — Sialic acid-interacting residues in 52SFK are shown on red background, together with similar, potential sialic acid-interacting residues of other AdV:s. Representative types have been selected from human species A-G and canine species A (HA-G and CA, respectively). Secondary-structure elements (beta strands) of HAdV-5 are indicated by arrows. (TIF) [file ppat.1004657.s010.tif]

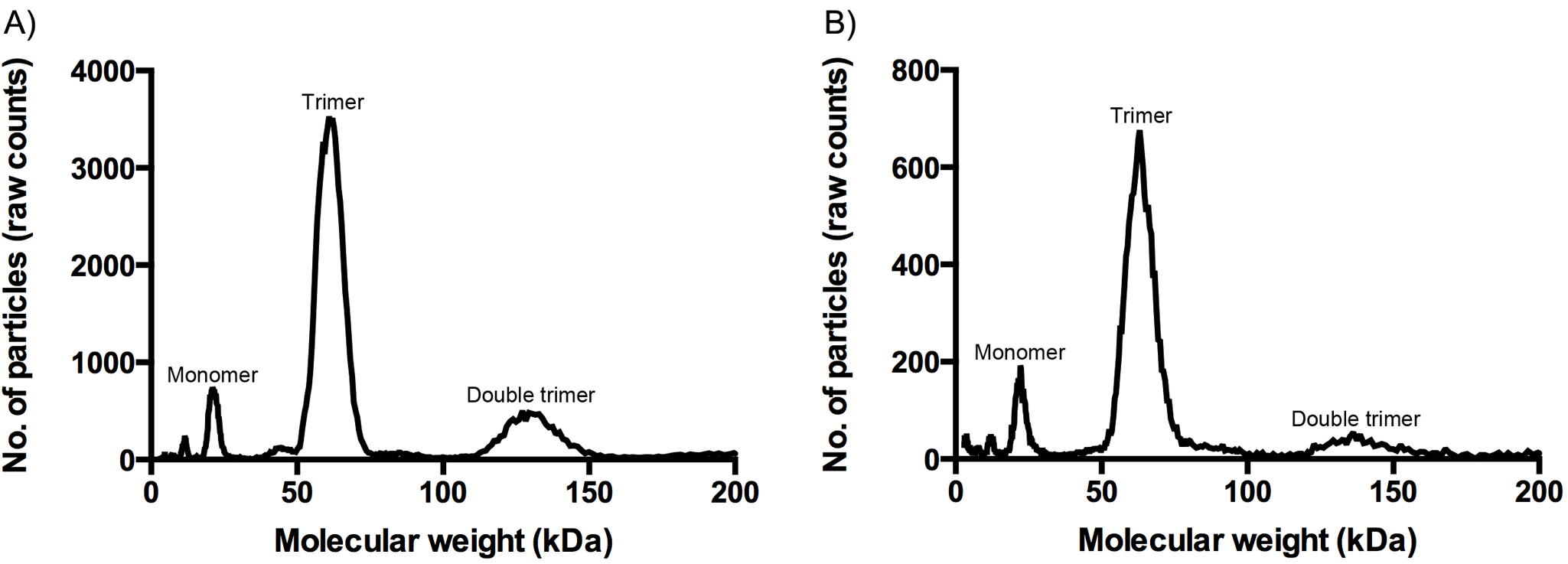

Supplement: S11 Fig — A) 52SFK wt and B) 52SFK R316A mutant were separated according to size using gas-phase electrophoretic mobility molecular analysis (GEMMA). Results are shown as number of molecules (particles) in respect to molecular weight (kDa). (TIF) [file ppat.1004657.s011.tif]

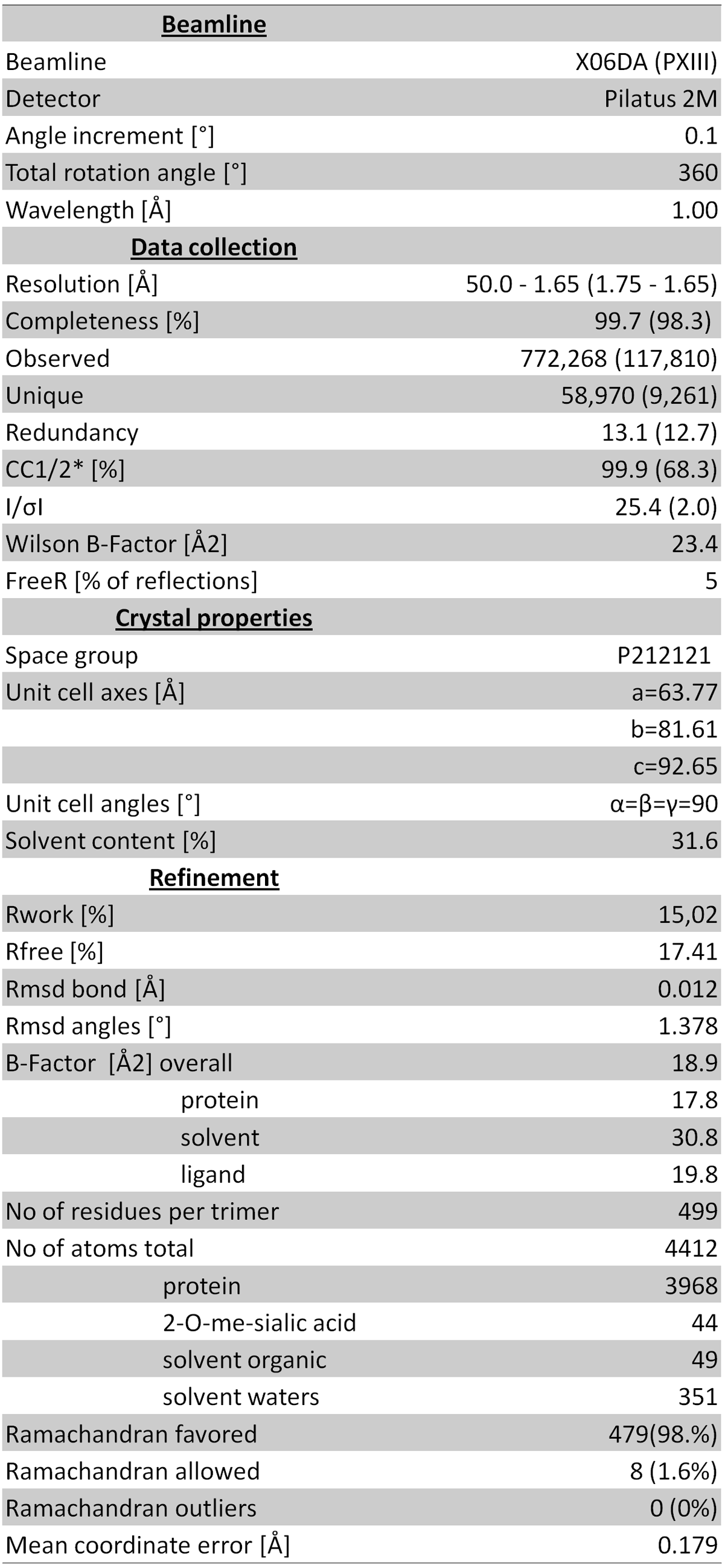

Supplement: S12 Fig — Values for the highest resolution shell are shown in parenthesis. (TIF) [file ppat.1004657.s012.tif]
